# Engineering of novel DNA polymerase variants for single enzyme quantitative multiplex reverse transcription-PCR

**DOI:** 10.1038/s41598-025-10211-x

**Published:** 2025-07-18

**Authors:** Luisa B. Huber, Virginie Marchand, Melike Somtürk, Silke Müller, Andreas Marx

**Affiliations:** 1 Department of Chemistry, Konstanz Research School Chemical Biology, University of Konstanz, Universitätsstraße 10, 78464 Konstanz, Germany; 2 SMP IBSLor, Epitranscriptomics and RNA Sequencing Core Facility, Université de Lorraine, Nancy, 54000 France; 3 Department of Chemistry, University of Konstanz, Universitätsstraße 10, 78464 Konstanz, Germany; 4 Department of Biology, Screening Centre, University of Konstanz, Universitätsstraße 10, 78464 Konstanz, Germany

**Keywords:** Biochemistry, Assay systems

## Abstract

Reverse transcription polymerase chain reaction (RT-PCR) has evolved as a widely used approach in biotechnology and molecular diagnostics. It represents a powerful tool for amplifying and analysing RNA molecules and has therefore found widespread applications in profiling gene expression, viral detection and the diagnosis of various diseases. Wellestablished methodologies use viral reverse transcriptases (RTs) to transcribe RNA to cDNA and thermostable DNA polymerases (DNA pols) to amplify the resulting target sequence by PCR. This study reports on the development of novel *Thermus aquaticus* DNA polymerase I (Taq pol) variants that each are able to catalyse both steps simultaneously in a single tube without the need of viral RTs. In combination with their excellent thermostability (up to 95 °C), the novel Taq pol variants are suitable for employment in dye- or probe-based RNA detection methods. Moreover, the herein reported Taq pol variants are capable of performing multiplex detection of various RNA targets in a single tube with a single enzyme. Thus, discovery marks a significant advancement of current RT-PCR approaches and contributes simplifying and reducing costs in molecular diagnostics.

**Supplementary Information:**

The online version contains supplementary material available at 10.1038/s41598-025-10211-x.

## Introduction

The combination of PCR with reverse transcription (RT) laid the basis for various powerful methods in the research field of molecular biotechnology and clinical diagnostics^1,2^. RT-PCR is used to detect and quantify even low-abundant RNA molecules and is therefore the gold standard in gene expression analysis, generating cDNA libraries and the detection of viral or pathogen infections^2–6^. Moreover, its excellent potential is applied for both cancer diagnosis and profiling and was recently coupled to high throughput next-generation RNA sequencing (NGS) technologies^7–9^. In particular, during the global pandemic caused by the severe acute respiratory syndrome coronavirus 2 (SARS-CoV-2), RT-PCR gained strong popularity and the importance of improving established techniques became apparent^10–12^.

Classical approaches start with the RT from RNA to DNA catalysed by viral RTs, followed by DNA purification and DNA amplification^2,13,14^. Either fluorescent dyes that intercalate into the DNA product or fluorescently tagged hydrolysis probes (so called TaqMan probes) are commonly used to monitor the reaction^15–17^. To simplify the procedure, RT and the subsequent DNA amplification were combined in a single tube formulation^18,19^. This not only saves time, but also reduces the risk of contaminations and eliminates the need to check buffer systems for compatibility^14,20^. Furthermore, the usage of different primer sets with distinctly labelled fluorescence probes enabled the simultaneous targeting of two or even multiple RNA regions in one reaction (multiplexed RT-PCR)^21–24^. This method offers substantial advantages such as saving time, reduced consumption of sample material and lower costs^25^. This technique is successfully used to profile mRNA levels from multiple target genes^26–29^ detect several viral strains, subtypes or other pathogens simultaneously^30,31^ and monitor cancer-related biomarkers for personalised medicine approaches^32,33^. To further enhance the assay performance, the applied enzymes should meet specific requirements. These include high catalytic efficiency at elevated reaction temperatures, good fidelity, enzymatic stability after multiple thermocycling steps, and the ability to produce even longer cDNA products^13^. Taking these criteria into account, both RTs and DNA pols have been continuously engineered and improved over the last years:

Viral RTs from Moloney murine leukaemia virus (M-MLV) and avian myeloblastosis virus (AMV) are commonly used for cDNA synthesis^13,14,34^ along with those from HIV-1 and bacterial group II introns^35,36^. A key challenge is their limited thermostability, even when RT precedes the PCR cycle^37^. Higher temperatures allow for a more specific priming process, make complex RNA or DNA structures accessible, and inactivate disruptive factors such as RNases^38,39^. Technical advances now allow cDNA synthesis at 37–60°C^13,40^ with some HIV-1 RT variants functioning at up to 85°C^41^. However, RT-PCR still requires thermostable DNA pols for amplification, and there is evidence that RT and DNA pols may compete for template binding, potentially affecting reaction efficiency, particularly at low RNA input^42^.

As an alternative, highly thermostable DNA pols with improved RT activity have been developed^43–48^. Especially, Taq pol naturally has RT activity, which can be enhanced under certain reaction conditions^49–51^. However, its application is limited by two major challenges: replacement of Mg²⁺ by Mn²⁺ increases RT activity but reduces fidelity^44,52^ or its low RT efficiency requires either high RNA input or buffer optimisation^49,53^. Protein engineering has further improved the RT activity of Taq pol or its truncated form and other DNA pols, thus single enzyme real-time RT-PCR was finally demonstrated^14,43,49,54–61^. However, many existing approaches still rely on Mn²⁺ additives or enzyme fusions^43,60^ lack compatibility with hydrolysis probes^54,55,57–59,61^, or have not been demonstrated in multiplex applications^49,56^. To expand these capabilities, we aimed to develop a streamlined TaqMan probe-based RT-PCR system that allows multiplex RNA targeting and is as easy to implement.

This study reports the development and application of novel DNA pol variants that are able to catalyse RT and the DNA amplification simultaneously without the need of viral RTs, addition of Mn^2+^ ions or to be fused to other enzyme scaffolds. These enzymes derived from the DNA polymerase I of *Thermus aquaticus* (Taq), which is known for its thermostability, high processivity, and ability to digest TaqMan probes^15,62–64^. The enzymes were obtained by combinatorically investigating two independently discovered mutation pools that enhance the RT activity of the *N*-terminally truncated form of Taq pol termed KlenTaq (KTq) DNA polymerase^57,61^. Promising variants were selected after screening the mutant library for RT and PCR activity. We could show that the application of those enzymes in RT-PCR further simplifies the one-tube formulation by eliminating the need for viral RTs as a second enzyme. In this way, we were able to perform RT-PCR by using either SYBR green I as intercalating fluorescent dye or a TaqMan probe-based approach. Moreover, we were able to detect up to four RNA targets simultaneously with a detection limit of 20 copies. This method marks a significant advancement, enabling the simultaneous detection of four RNA targets through quadruplex RT-PCR, all achieved through the utilization of a single enzyme capable of catalysing both RT and DNA amplification.

## Results

### Screening for Taq pol variants with improved RT-PCR activity

**Fig. 1:**
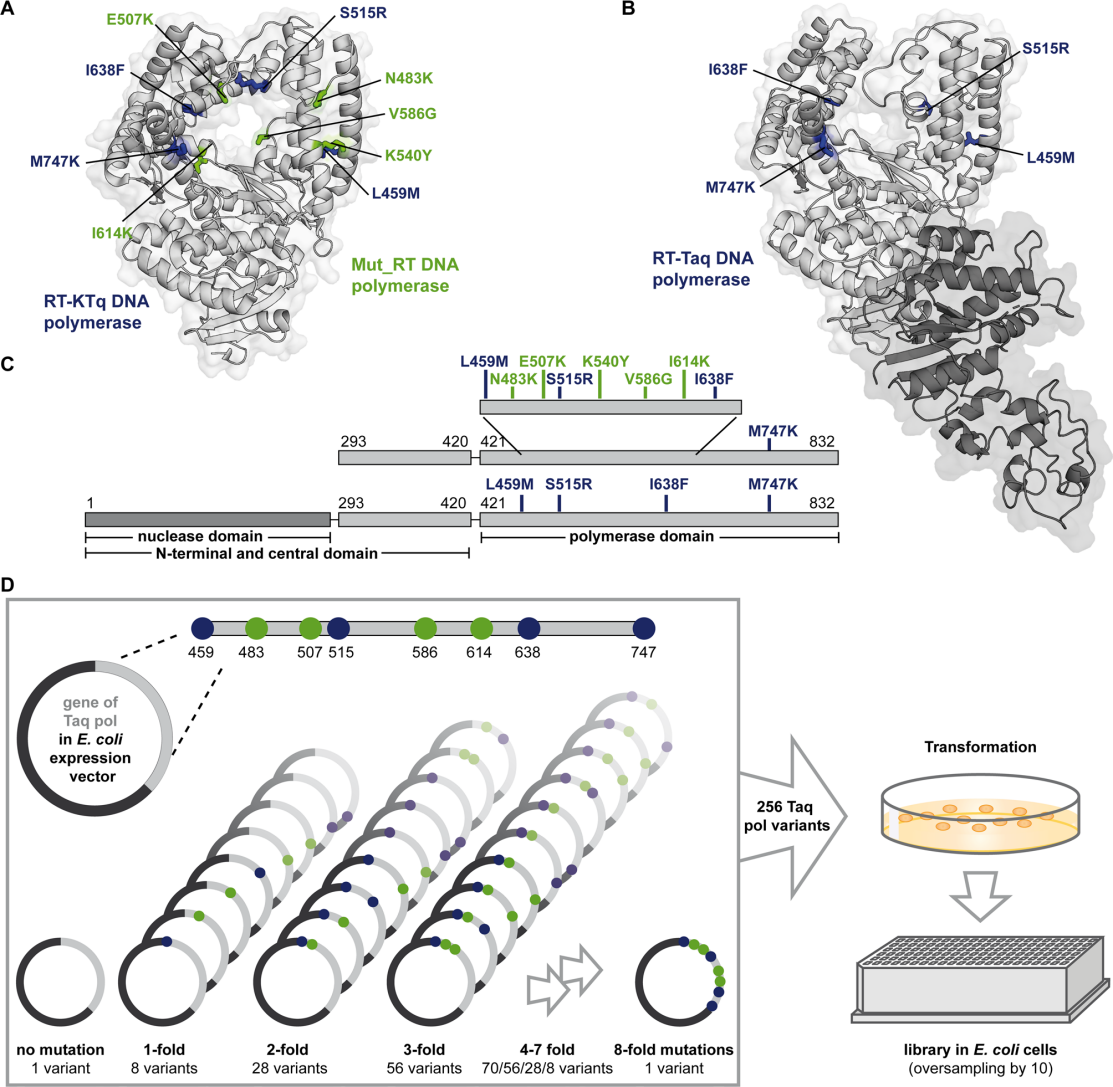
Crystal structures of RT-KTq/Mut_RT and Taq pol. (**A**) The overall crystal structure of RT-KTq and Mut_RT is shown as cartoon (adapted from PDB ID: 4BWM using PyMOL (Schrödinger, LLC, New York, NY)). Amino acid mutations specific for RT-KTq are coloured in blue and mutations specific for Mut_RT are coloured in green (side chains depicted as sticks). The KTq pol backbone is coloured in light grey. (**B**) The overall crystal structure of Taq pol is shown as cartoon (adapted from PDB ID: 1TAQ using PyMOL (Schrödinger, LLC, New York, NY)). The KTq pol backbone is coloured in light grey and the nuclease domain is depicted in dark grey. The amino acids that are changed to form the RT-Taq are coloured in blue. (**C**) Architecture of the KTq pol variants (top) and Taq pol (bottom). The positions of amino acid mutations are indicated in blue (RT-KTq, RT-Taq) and green (Mut_RT). (**D**) Combination strategy of the new Taq pol enzyme library. All eight amino acid mutations were combined to generate the wild-type Taq pol (without mutations), all single, double, triple and 4 − 8 fold mutants giving 256 Taq pol variants in total. The plasmid mix was transformed into *E. coli* cells, plated and 2660 single colonies were picked to form the library.

Previous studies have identified two mutational pools from two previously engineered KTq pol variants: the RT-active KTq DNA polymerase (RT-KTq)^57^ and Mut_RT DNA polymerase (Mut_RT)^61^. RT-KTq carries 4 amino acid mutations namely L459M, S515R, I638F and M747K (Fig. 1A and C, amino acid side chains depicted in blue)^57^. The enzyme was obtained by a recombined DNA shuffling approach that combined seven distinct mutations previously reported to promote either RT or to expand the substrate spectrum^55,65^. After pre-selection for both PCR- and RT activity, RT-KTq showed up to 100-fold increased RT compared to the applied parental enzymes. Structural analysis of RT-KTq suggested that mutations such as L459M and S515R enhance thumb domain flexibility, while I638F and M747K may stabilize substrate binding through hydrophobic or electrostatic interactions^57^.

In contrast, a combined rational and combinatorial enzyme library design revealed Mut_RT, carrying five mutations, namely N483K, E507K, K540Y, V586G and I614K (Fig. 1A and C, amino acid side chains depicted in green)^61^. This enzyme showed significantly increased activity on both RNA and DNA compared to the parental enzyme. Although the Mut_RT mutations were not structurally analysed, they were selected due to their proximity to the primer/template complex and their evolutionary conservation among family A DNA pols, suggesting a role in substrate interaction and catalytic efficiency^61^.

Taken together, RT-KTq and Mut_RT originated from two independently constructed enzyme libraries using different approaches. As consequence, two different pools of mutations were reported to successfully increase RT-activity. We hypothesized that recombining proficient mutations from both enzymes might result in an even more empowered RT-active DNA pol. In addition, since the full-length Taq DNA pol was shown to be more processive than its truncated version^64,66^ and insertion of RT-boosting mutations into the Taq pol backbone increased RT-activity compared to its respective KTq pol variant^56^ we used this scaffold. Thus, we introduced the 4 mutations of RT-KTq into the Taq pol backbone to form the RT-Taq (Fig. 1B and C bottom, amino acid side chains depicted in blue).

In preliminary experiments, RT-KTq and all 5 single mutants of the Mut_RT were tested individually to evaluate their contributions to enzyme performance. Not all mutation sites contributed equally: KTq pol K540Y showed neither primer extension nor RT-PCR activity (Supplementary Fig. S1, A, B, C, Supplementary Fig. S8, Supplementary Fig. S9 A). As consequence, this mutation was excluded from the following screening approach. However, the experiment demonstrated that the combination of different mutations in Mut_RT resulted in positive synergistic effects, which together improve the enzyme performance.

Based on these findings, a new enzyme library of Taq pol mutants was designed to recombine the differently evolved RT-enhancing mutation pools. The library included all possible combinations of L459M, N483K, E507K, S515R, V586G, I614K, I638F and M747K with the respective wild-type amino acids. Thus, the library comprised the wild-type Taq pol without mutations, as well as all single, double, triple and 4 − 8 fold mutants, resulting in a total of 256 Taq pol variants (Fig. 1D). In this way, the RT-Taq and the Taq pol variant bearing the Mut_RT specific mutations were included, as well. The genes of all DNA pol variants were synthesised in equimolar amounts, inserted into the pGDR11 vector, and used as a mixture. The plasmid mix was transformed in *E. coli* BL21 (DE3) cells and plated on agar plates. A total of 2660 single colonies were picked and arranged in a 348well format. Oversampling by a factor of more than 10 during colony picking ensured that more than 99% of all possible Taq pol variants were covered and included in the library^67^. Cell lysates from expression cultures were directly used after heat inactivation of *E. coli* host proteins.

**Fig. 2:**
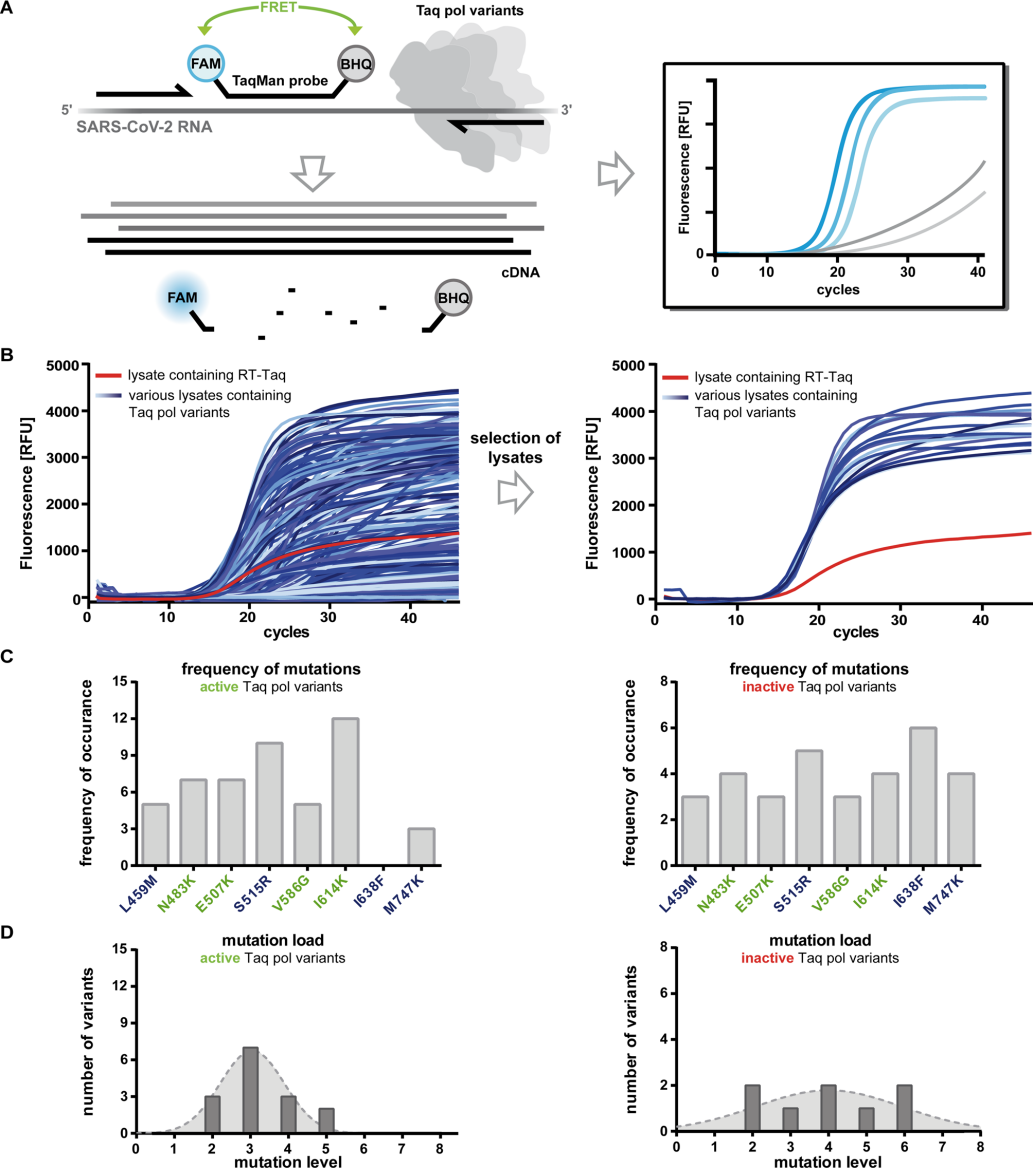
Screening approach and selection for identifying Taq pol variants with increased RT-PCR activity (**A**) Principle of the screening assay. RT and amplification of the cDNA is catalysed by different Taq pol variants. The applied TaqMan probe is 5’- fluorescently labelled with FAM (6-carboxyfluorescein) and 3’- labelled with BHQ1 (Black Hole Quencher 1). Product formation can be followed by monitoring the increase in fluorescence signal as the probe is digested by the Taq pol variants and fluorescence is no longer quenched by BHQ1. (**B**) Selection of promising Taq pol variants are shown as an example for the first expression plate (amplification curve for lysate containing the RT-Taq depicted in red and amplification curve for lysates containing different Taq pol variants depicted in blue). Selection of promising variants were based on lower Cq values than those of the RT-Taq (red curve) and amplification curves following a sigmoidal shape. (**C**) Frequency of each mutation (L459M, N483K, E507K, S515R, V586G, I614K, I638F and M747K) is depicted in active vs. inactive Taq pol variants. Left: 15 different Taq pol variants were included in the group of active variants and right: 8 Taq pol variants were included in the group of inactive variants. (**B**) Mutation level (0-fold, 1-fold, 2-fold…) is plotted against their frequency. Left: 15 different Taq pol variants were included in the group of active variants and right: 8 Taq pol variants were included in the group of inactive variants.

The screen comprised a real-time RT-PCR approach that was previously established to detect the SARS-CoV-2 RNA in unprocessed swab samples^10^. Primers and TaqMan probes used in the study were similar to those applied in the FDA emergency-use authorized CDC (Centers for Disease Control and Prevention) diagnostic assay and target the nucleocapsid gene N1^68^. After the precise and efficient PCR, a 72 bp DNA amplicon was generated. The SARS-CoV-2 RNA was ordered from the European Commission Joint Research Centre (CRM code: EURM-019) and included in vitro transcribed single-stranded RNA from the SARS-CoV-2. The formation of the cDNA could be followed by monitoring the increase in fluorescence signal since the hydrolysis probe is digested by the Taq pol during the ongoing PCR, leaving the unquenched FAM (6-carboxyfluorescein) dye in the reaction mix (Fig. 2A).

The reaction conditions were optimised for the use of expression lysates and the RNA template concentration of 1000 c/µL (copies/µL) was found to be sufficient to adequately monitor the enzyme performance (Supplementary Fig. S2). After screening 2660 cell lysates, promising Taq pol variants were selected based on lower Cq values than those of the RT-Taq and amplification curves following a sigmoidal shape (Fig. 2B).

The best 379 variants were selected and corresponding cells were transferred to a new 384 deep well plate. Five positions on the plate were occupied by *E. coli* cells carrying the RT-Taq vector. The cell lysates were screened again by applying a RNA template dilution from 1000 − 1 c/µL and different forward primer to generate PCR products with increasing lengths (72, 100, 156, 208 and 254 bp). Finally, 20 Taq pol variants showed good performance in all reaction conditions (Supplementary Fig. S3) and 46 variants performed well in only 8 from 9 conditions. The expression levels were checked for uniformity by SDS-PAGE (Supplementary Fig. S4 A, Supplementary Fig. S9 B, C, Supplementary Fig. S10 A). In order to determine the mutation patterns of the 20 most promising Taq pol variants, plasmids from corresponding *E. coli* cells were isolated and sent for Sanger sequencing. 15 different DNA genes were found since some *E. coli* cells carried the same plasmid encoding for the same Taq pol variant (Table 1).

**Table 1:**
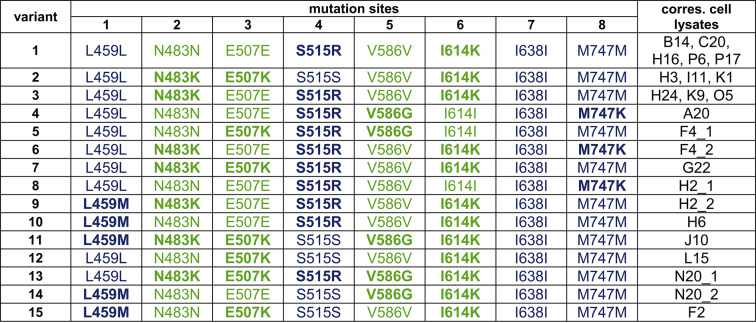
Composition of the mutation sites found in the 20 most promising Taq pol variants. Corresponding (corres.) cell lysates are indicated. Mutation sites originate from RT-KTq are coloured in blue and mutation sites originate from mut_rt are coloured in green. Positions with mutations are highlighted in bold letters.

Moreover, some plasmids from *E. coli* cells whose lysates showed no activity were also analysed by Sanger sequencing (Table 2). To ensure that lower activity was not due to low expression levels, only those lysates were selected that show protein expression upon SDS-PAGE analysis (Supplementary Fig. S4 B, Supplementary Fig. S10).

**Table 2:**
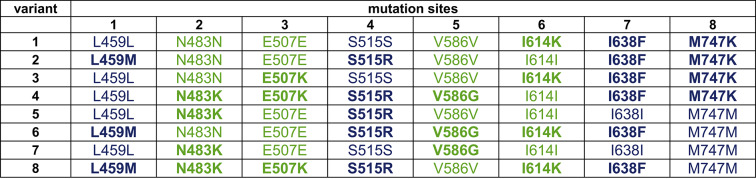
Composition of the mutation sites found in inactive Taq pol variants. Composition of the mutation sites found in inactive Taq pol variants. Mutation sites originate from RT-KTq are coloured in blue and mutation sites originate from Mut_RT are coloured in green. Positions with mutations are highlighted in bold letters.

When comparing the mutation composition of active and inactive Taq pol variants, it is striking that the mutation I638F only occurs in the inactive variants (Fig. 2C). I614K was the most common mutation in the active variants, while it was less present in the inactive ones. Interestingly, most of the well performing variants carried only three mutations in their amino acid sequence (Fig. 2D, left). However, within the group of inactive variants, there were some enzymes with a higher mutation load (Fig. 2D, right). These two aspects indicate that not only the composition of RT-promoting mutations, but also a moderate mutation load of about three mutations contributes to a well-performing enzyme.

### Further characterization of promising Taq pol variants

**Fig. 3:**
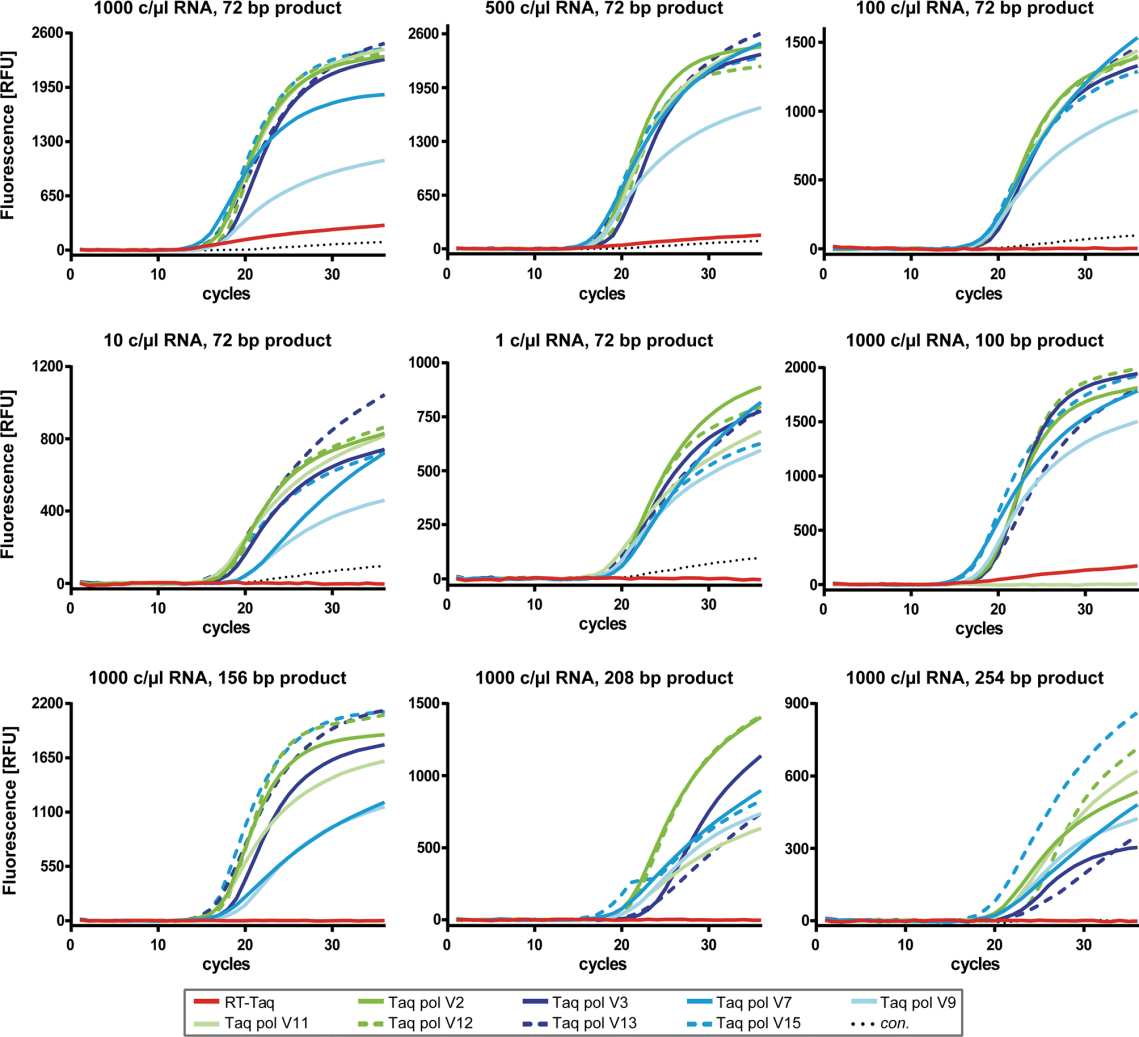
Amplification curves after RT-PCR from SARS-CoV-2 RNA catalysed by purified Taq pol variants. 30 nM enzyme, 100 nM Taq pol aptamer, 670 nM primer, 170 nM TaqMan probe and SARS-CoV-2 RNA (concentrations as indicated) were present in the reaction mix. Forward primer was varied to generate PCR products with different lengths (as indicated). The same mastermix was used for the control reaction (con.), but the amount of RNA template was replaced by Milli-Q water. The reactions with diluted RNA template were performed twice and reactions forming longer amplicons were conducted once.

For further studies, all 15 promising RT-active Taq pol variants were purified and subjected to a similar screen as described above for the study of the most promising *E. coli* cell lysates. For this purpose, we used a RNA template dilution from 1000 − 1 c/µL to further increase the stringency of the assay conditions. Furthermore, RT-PCR was performed with different forward primers to generate PCR products of increasing length (72, 100, 156, 208 and 254 bp). Of note, the same reaction conditions were selected for all approaches without any further optimisation, even if there was low input of RNA or longer amplicons had to be generated. Taq pol V2, V3, V7, V9, V11, V12, V13 and V15 showed the best amplification curves over all conditions (Fig. 3).

Product formation was observed even in more demanding reactions, such as those with very low RNA template input or the synthesis of longer amplicons. Similar to reactions performed with *E. coli* lysates, RT-Taq was not able to amplify from RNA templates with low concentrations and to form longer PCR products (Fig. 3, red curves).

**Fig. 4:**
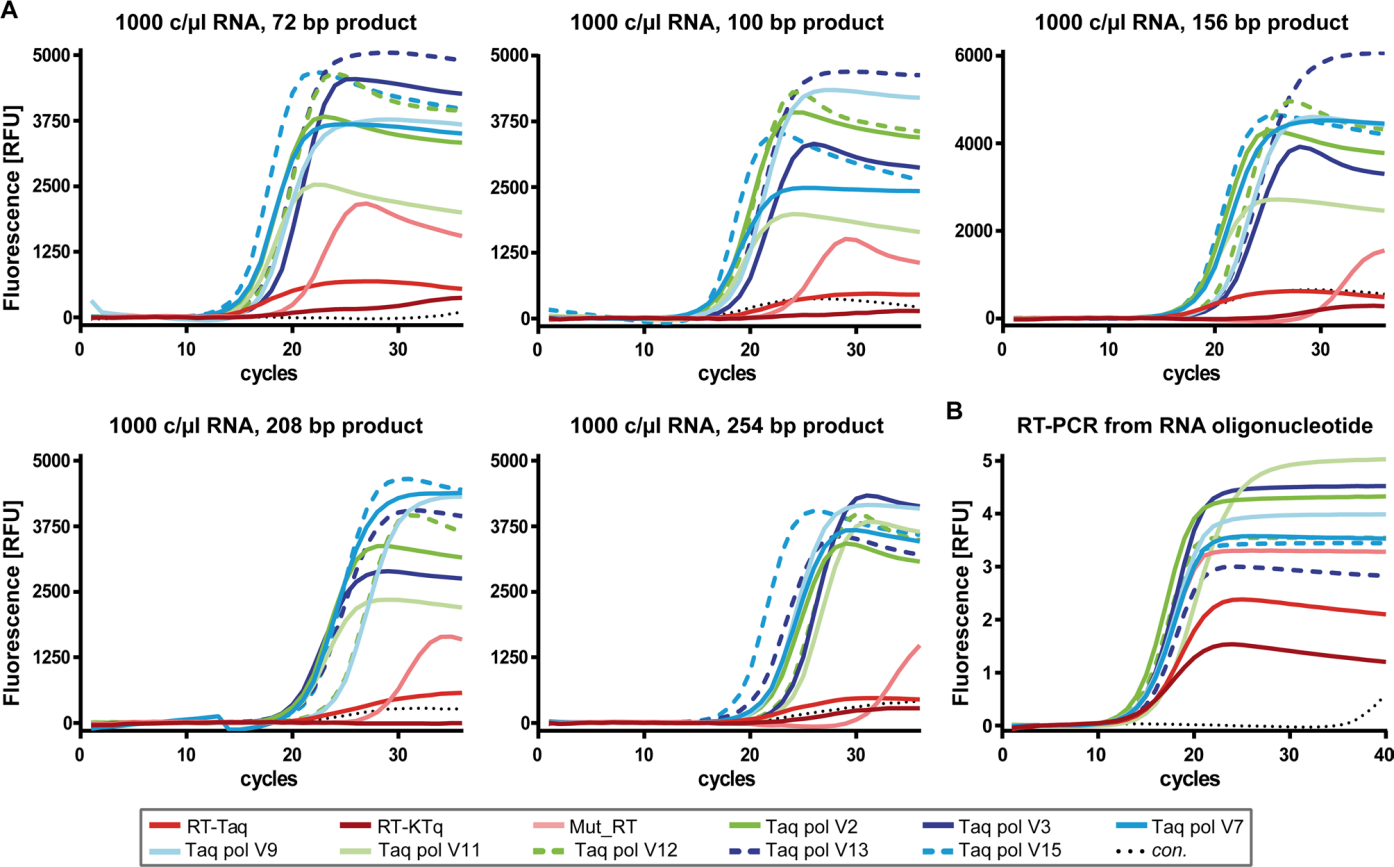
Amplification curves after RT-PCR from SARS-CoV-2 RNA and from the artificial RNA used in preliminary studies. (**A**) 1000 c/µL SARS-CoV-2 RNA was used as template and different forward primers were applied to generate PCR products of different lengths (as indicated). 30 nM purified Taq pol variants (as indicated) and 100 nM Taq pol aptamer was used for catalysis. The reaction was monitored by SYBR green I intercalating into the forming cDNA. (**B**) 100 nM artificial RNA template was present in the reaction. 100 nM purified Taq pol variants (as indicated) and 100 nM Taq pol aptamer was used for catalysis. The reaction was monitored by SYBR green I intercalating into the forming cDNA. **A**)/**B**) For the control reaction (con.), the same mastermix was used but the amount of RNA template was replaced by Milli-Q water. The reactions from the SARS-CoV-2 RNA template were performed twice and reactions from the artificial RNA template were conducted three times.

Next, we investigated if the identified Taq pol variants tolerate SYBR green I in real-time PCR. Therefore, all 15 Taq pol variants were applied again in the RT-PCR assay using SARS-CoV-2 RNA with different combinations of reverse and forward primers. Similar to observations above Taq pol V2, V3, V7, V9, V11, V12, V13 and V15 showed the best amplification curves over all reactions (Fig. 4A). Furthermore, the experiment showed that the improved RT-PCR activity of the identified Taq pol variants is not only due to a higher nuclease activity, but most importantly due to an improved PCR activity.

In order to examine whether the purified Taq pol variants can catalyse RT-PCR without a prior RT step, we performed amplification from an RNA oligonucleotide under time-limited conditions. Here, the cDNA was obtained by direct heating to 95°C and subsequent conducting a two-step amplification. For comparison, the RT-KTq, Mut_RT and RT-Taq and all 15 Taq pol variants were tested using the same reaction parameters as shown in Supplementary Fig. S1 B. The amplification curves measured for Taq pol V2, V3, V7, V9, V12 and V15 passed the threshold at the same time or earlier than RT-KTq, Mut_RT or RT-Taq (Fig. 4B). The highest activity was observed for Taq pol V2 and V3 starting the DNA amplification after a low number of cycles and generating a high fluorescence intensity. Indeed, this experiment demonstrated that the most effective Taq pol variants identified in the screening did not require an additional RT step prior to amplification. Considering all RT-PCR experiments with purified enzyme, Taq pol V2, V3, V12 and V15 showed the best performance in terms of low Cq values and amplification curves with high fluorescence intensities.

### Taq pol variants are able to catalyse multiplexed RT-PCR from the SARS-CoV-2 RNA

Having established that Taq pol V2, V3, V12, V15 can faithfully catalyse RT-PCR from the SARS-CoV-2 RNA targeting the nCov_N1 gene, we investigated whether other targets could be detected using this approach. We performed RT-PCR using the primer and probe sets for the nCov_N2 and nCov_E genes that correspond to the FDA-approved CDC emergency diagnostic assay^68^. Initially, all gene targets were monitored individually in order to find a suitable reaction condition for all of them. Moreover, we have introduced the additional mutation I707L into the backbone of the Taq pol V2 (henceforth referred to as Taq pol V2 IL) to investigate whether this can further improve PCR activity. Barnes and colleagues had shown that I707L reduce the polymerase activity at lower temperatures, while retaining normal activity at the optimal reaction temperature of the KTq pol enzyme^69^. Taq pol V2 were selected as an example because of its excellent performance in previous experiments.

**Fig. 5:**
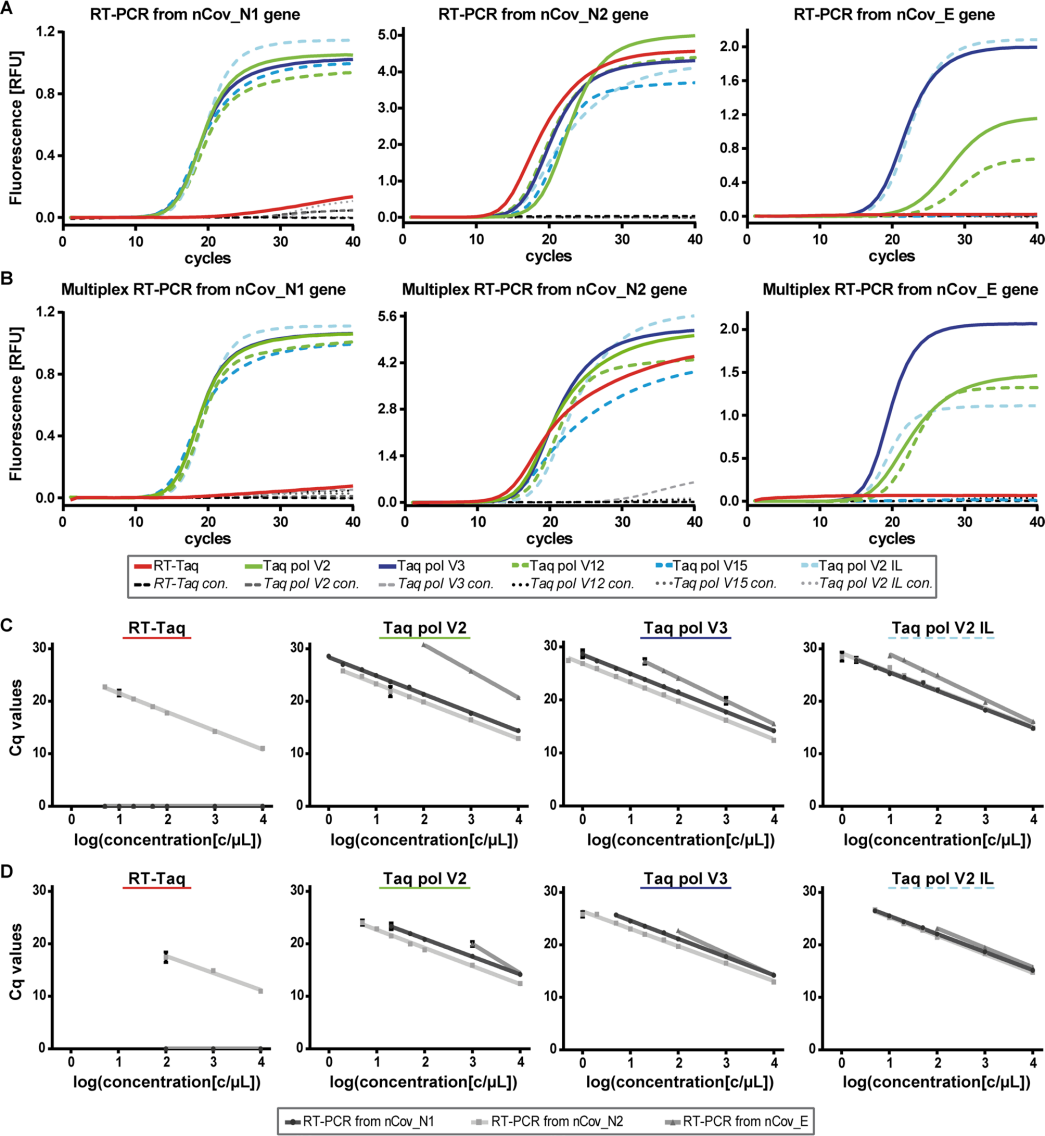
Amplification curves after RT-PCR from SARS-CoV-2 RNA in singleplex and multiplex reaction set-up. (**A**) 10.000 c/µL SARS-CoV-2 RNA was used as template and the genes nCoV_N1, N2 and E were analysed independently (as indicated). 120 nM purified Taq pol variants (as indicated) and 400 nM Taq pol aptamer was used for catalysis. 670 nM primer, 170 nM TaqMan probe (in case of nCoV_N1/N2) or 850 nM primer, 216 nM TaqMan probe (in case of nCov_E) were present in the reaction mix. Reactions were monitored by measuring the fluorescence from the 6-FAM dye (nCoV_N1), the Sun dye (nCoV_N2) and the Texas Red dye (nCov_E). (**B**) 10.000 c/µL SARS-CoV-2 RNA was used as template and the genes nCoV_N1, N2 and E were analysed simultaneously (triplex RT-PCR). The reaction conditions were similar to reactions described in A), but all primer sets were present in the same reaction. (**C**) SARS-CoV-2 RNA template was diluted stepwise and the genes nCoV_N1, N2 and E were analysed independently (singleplex RT-PCR). (**D**) SARS-CoV-2 RNA template was diluted stepwise and the genes nCoV_N1, N2 and E were analysed simultaneously (triplex RT-PCR). C)/D) The same reaction conditions were use as in **A**) or **B**). Respective average Cq values were plotted against the decadic logarithm of the template concentration. Linear regression was applied. Linear functions, R^2^ values and the PCR efficiencies are indicated in Supplementary Table S4. Error bars indicating the standard deviation of duplicates are shown in black. **A**)/**B**)/**C**)/**D**) The same mastermix was used for the control reaction (con.), but the amount of RNA template was replaced by water. Reactions were performed in duplicates.

All variants of the Taq pol were able to catalyse the reaction from each SARS-CoV-2 gene under the same optimised reaction condition (Fig. 5A). Taq pol V15 is partially excluded here, as it did not show an increase in the fluorescence signal for the Texas Red dye and thus detection of the nCov_E gene was not successful. The parental enzyme RT-Taq was only able to amplify products from the nCov_N2 gene and to a much lesser extent from the nCov_N1 gene (Fig. 5A, red curves). Of note, detecting the nCov_E gene is more challenging than detecting the other targets because the other amplicons are shorter in length (72 bp for nCov_N1, 67 bp for nCov_N2 and 113 bp for nCov_E). This experiment benefited from the exceptional thermostability of the enzymes since RT was carried out at 65 °C.

Next, we challenged the Taq pol variants by gradually reducing the template concentration to determine the limit of detection. With this reaction setup, it should be noted that uniform reaction conditions were defined for all genes to allow simultaneous detection. Consequently, the conditions for nCoV_E were not individually optimised. Since the Taq pol V2 IL had previously shown an improved performance on the nCov_E gene compared to the Taq pol V2, this variant was considered further. The Cq values were plotted against the decadic logarithm of the template concentration and linear regression was applied (Fig. 5C, Supplementary Table S4). The parental RT-Taq pol was able to detect 100 copies of the SARS‑CoV‑2 template (5 c/µL) by monitoring the nCov_N2 gene. The dilution series was amplified with a PCR efficiency of 91%. Other targets could not be detected adequately (Fig. 5C, left). Taq pol V2, V3 and V2 IL showed the lowest detection limits with 20, 10 and 20 copies respectively (Fig. 5C, three plots right). All dilution series had a PCR efficiency above 90% except for the reaction from the nCov_E gene. These results render Taq pol V2, V3 and V2 IL as promising candidates.

In the next step, we investigated if the nCov_N1, nCov_N2 and the nCov_E gene could be monitored simultaneously in one reaction. The promising Taq pol variants V2, V3, V12, V15 and V2 IL were applied in multiplexed RT-PCR under similar conditions as in the singleplex reaction setup (Fig. 5B). At a template concentration of 10.000 c/µL, all DNA pols, except Taq pol V15 and the parental RT-Taq, successfully amplified all three genes. As in the previous experiments, the concentration of the SARS-CoV-2 template was gradually decreased, the corresponding Cq values were plotted against the decadic logarithm of the template concentration and linear regression was applied (Fig. 5D, Supplementary Table S4).

When monitoring the nCov_N2 gene, the parental RT-Taq showed a reduced sensitivity from 100 (singleplex) to 2000 copies (multiplex). PCR efficiency was determined to be 104%. However, Taq pol V2 and V2 IL were still able to detect 100 copies, while Taq pol V3 detected only 20 copies of RNA. Thus, the latter is 100 times more sensitive than the parental RT-Taq. The PCR efficiencies are consistently above 92%, with the exception of the reactions from the nCov_E gene (Supplementary Table S4). Overall, this demonstrates that Taq pol V2, V3 and V2 IL are able to catalyse RT-PCR in singleplex and multiplex reaction formulations and independently of the RNA target sequence.

We then tested whether the amplification from the human RNase P transcript could be used as an internal control reaction to verify the integrity of the patient samples in the future. For this purpose, RT-Taq, Taq pol V2, V3 and V2 IL were used in multiplexed RT-PCR targeting the SARS-CoV-2 nCov_N1, nCov_N2, nCov_E gene and the RNase P transcript simultaneously (quadruplex RT-PCR). The universal human reference RNA (Thermo Fisher Scientific) was used as template and the primer/probe sets from the FDA authorized CDC diagnostic assay were applied for catalysis^68^.

**Fig. 6:**
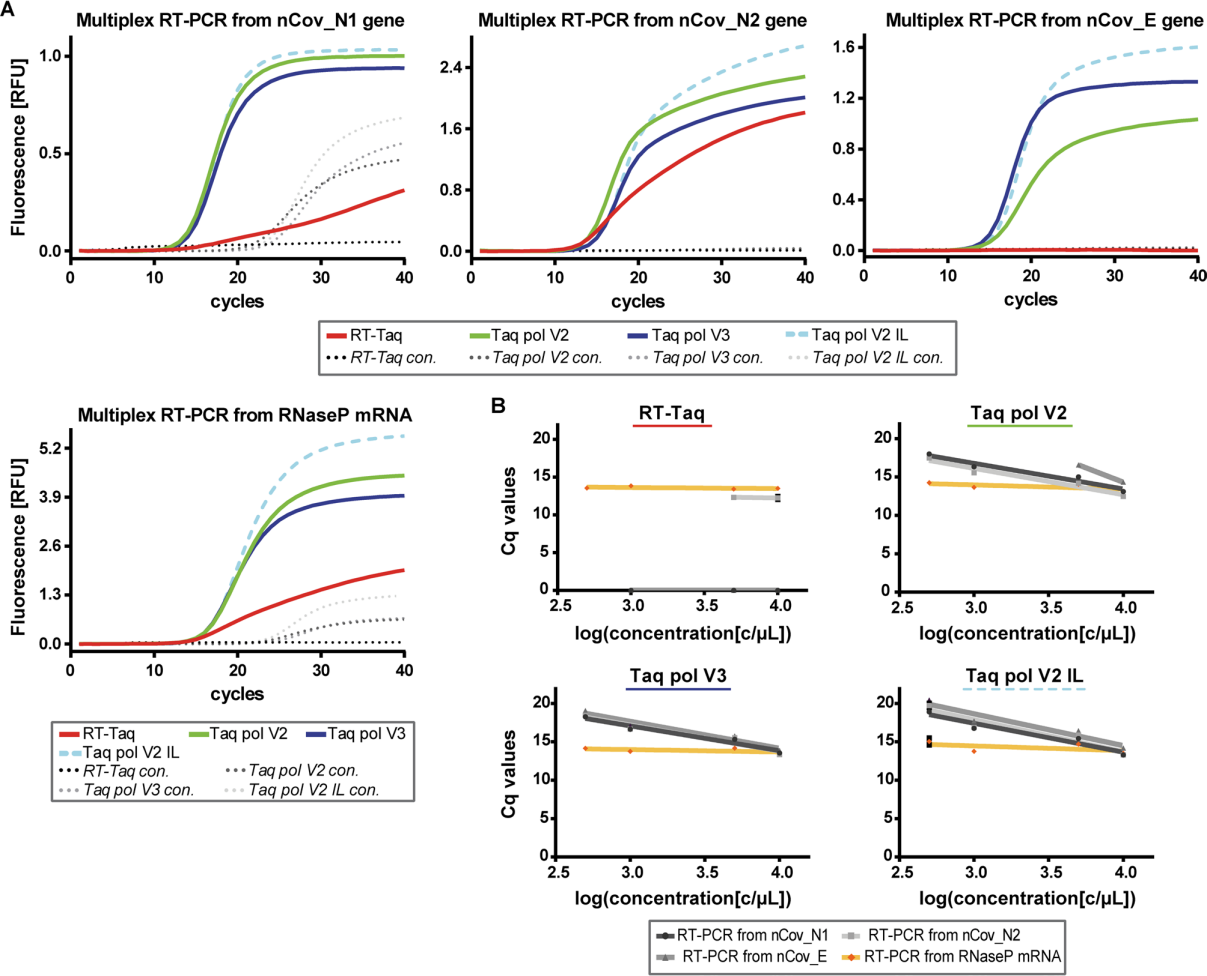
Amplification curves after multiplex RT-PCR from SARS-CoV-2 RNA and human RNase P transcript. (**A**) 10.000 c/µL SARS-CoV-2 RNA was used as template. 1 ng/µL universal human reference RNA was used to monitor amplification from the RNase P transcript. The genes nCoV_N1, N2 and E, as well as the RNase P transcript were analysed simultaneously (quadruplex RT-PCR). 120 nM purified Taq pol variants (as indicated) and 400 nM Taq pol aptamer was used for catalysis. 670 nM primer, 170 nM TaqMan probe (for nCoV_N1/N2, RNase P) and 850 nM primer, 216 nM TaqMan probe (for nCov_E) were present in the reaction mix. Reactions were monitored by measuring the fluorescence from the 6-FAM dye (nCoV_N1), the Sun dye (nCoV_N2), the Texas Red dye (nCov_E) and the Cy5 dye (RNase P). (**B**) SARS-CoV-2 RNA template was diluted stepwise while keeping the human reference RNA constant. The genes nCoV_N1, N2, E and RNase P transcript were analysed simultaneously (quadruplex RT-PCR). Respective average Cq values were plotted against the decadic logarithm of the template concentration. Linear regression was applied. Linear functions, R^2^ values and the PCR efficiencies are indicated in Supplementary Table S4. Error bars indicating the standard deviation of duplicates are shown in black. A)/B) The same mastermix was used for the control reaction (con.), but the amount of RNA template was replaced by water. Reactions were performed in duplicates.

At the template concentrations of 10.000 c/µL of SARS-CoV-2 and 1 ng/µL human reference RNA, all DNA pols were able to successfully amplify the 4 target sequences (Fig. 6A). However, the parental RT-Taq was only able to catalyse the reaction from the nCov_N2 gene and the RNase P transcript (Fig. 6A, red curves). Of note, the reaction conditions for the quadruplex reaction did not need to be optimized again.

To further investigate the enzyme performance, we diluted the SARS-CoV-2 RNA to 500 c/µL in 4 steps, while keeping the human reference RNA constant at 1 ng/µL. The respective Cq values were plotted against the decadic logarithm of the template concentration and linear regression was applied (Fig. 6B, Supplementary Table S4). The best performance was observed for Taq pol V3, as all 4 RNA targets were successfully monitored at each dilution step and PCR efficiencies ranged from 93 to 104%. Furthermore, the Cq values for reactions from the RNase P transcript remained constant at approximately 13.9. The successful detection of SARS-CoV-2 observed in the triplex RT-PCR was therefore also achieved in the presence of a different RNA target, demonstrating the potential applicability of an internal standard in the reaction.

Taken together, we were able to demonstrate that the newly developed Taq pol variants can be successfully used in single- tube RT-PCR approaches without the need for a second enzyme. As the human reference RNA used in this study is a mixture of RNA extracts from 10 cell lines, SARS-CoV-2 was successfully detected from a complex mixture of RNA. Moreover, our data shows an example of a one-step quadruplex RT-PCR using only one single DNA polymerase without the addition of manganese ions.

Furthermore, we investigated the polymerase fidelity within the RT-PCR process. Therefore, the parental enzymes RT-Taq, Mut_RT, Taq pol V2, V3 and V2 IL were used to reverse transcribe and amplify a 100 nt segment of the nCov_N1 gene and the resulting amplicons were subsequently subjected to NGS (Supplementary Fig. S5). RT-Taq and Mut_RT showed comparable error rates and revealed an average error of 0.07%. Taq pol V2, V3 and V2 showed slightly increased error patterns compared to the parental enzymes resulting in average errors of 0.21%, 0.19%, and 0.14%, respectively. It should be noted that the KTq pol generally has a higher accuracy than the full-length Taq pol^64^. The introduction of the I707L mutation into the Taq V2 pol variant increased the polymerase fidelity by a factor of 1.5. Of note, naturally occurring RTs are known to have lower fidelity than DNA-dependent DNA polymerases^13^. Therefore, the catalysis during RT-PCR is likely to be more error prone. The Taq pol variants used in this study are DNA pols capable of accepting and processing RNA while successfully amplifying DNA. Thus, errors might not be primarily introduced in the first linear RT step, but also during amplification. Examination of the error distribution for each nucleobase identity separately showed that all DNA pols had the highest error rates for U, followed by A (Supplementary Fig. S6). The highest fidelity was observed for the incorporation of nucleotides opposite G and C bases.

Finally, the best working enzymes should be compared to a commercial two enzyme one-step RTPCR system. Here, the Luna^®^ Universal Probe One-Step RT-PCR Kit from NEB was used to catalyze the quadruplex RT-PCR from the SARS-CoV-2 RNA and the human RNaseP mRNA transcript by using the same primer and probes as described above. The reaction conditions and the cycling protocol were optimized according to the manufacture’s recommendations. For better comparability, Taq pol V2, V3, V2 IL, and RT-Taq were tested under the same reaction conditions optimised for the Luna^®^ Universal Probe One-Step RT-PCR Kit. The enzyme concentrations of the Taq pol variants were consistent with those in the previous quadruplex RT-PCR experiment. Luna enzymes were used in the amounts specified in the manufacturer’s protocol. However, as the supplier does not provide specific information on enzyme concentration or quantity, direct comparability with the Taq pol variants is limited in this respect. All reactions were carried out in the reaction buffer from the Luna^®^ Kit to ensure identical buffer compositions. Taq pol V2 and V3 showed comparable RT-PCR performances as the two enzyme system from the Luna^®^ Universal Probe One-Step RT-PCR Kit targeting all three SARS-CoV-2 genes and the human RNaseP transcript (Supplementary Fig. S7). Taq pol V2 IL showed a slight increase in Cq levels in experiments using the SARS-CoV-2 RNA as template compared to Taq pol V2 and V3. In contrast, RT-Taq showed no activity in processing the SARS-CoV-2 nCov_E gene and demonstrated lower RT-PCR performance in amplifying from the human RNase P transcript compared to the other enzymes. Of note, all Taq pol variants were functional under the buffer and reaction conditions of the Luna^®^ Universal Probe One-Step RT-PCR Kit, and optimising their enzyme concentration might further enhance performance.

## Discussion

In this study, we report on the development of RT-PCR active DNA polymerases, Taq pol V2, V3 and V2 IL, that differ by only 3 − 4 amino acids from wild-type Taq pol. The enzymes are capable of catalysing one-step RT-PCR. Moreover, these DNA pols are also capable of performing multiplex RT-PCR and thereby represent enzymes that are capable of performing multiplex RT-PCR with a single enzyme without the addition of manganese ions. Their use in well-established RNA detection approaches can not only improve current laboratory procedures in terms of time and cost, but also offers the possibility of addressing complex RNA targets due to their high thermostability. The Taq pol variants were discovered in a screening approach that combined two independently discovered pools of mutations enhancing the RT activity of the parental KTq pol^57,61^. The full length Taq pol was used as library scaffold because it is known to be more processive than its truncated version^56,64,66^. Moreover, the Taq pol scaffold allows their employment in hydrolysis probe-based test systems that are widely used in molecular diagnostics^17^. Several DNA pol variants were identified that are capable of detecting the exemplary chosen SARS-CoV-2 genome in the N1 region, even when the RNA template was diluted and the enzyme had to generate longer DNA amplicons. Further analysis and characterisation lead to the identification of Taq pol V2, V3 and V2 IL carrying the mutations N483K, E507K, I614K (Taq pol V2); N483K, S515R, I614K (Taq pol V3); and N483K, E507K, I614K, I707L (Taq pol V2 IL). These enzymes were able to detect the N1, N2 and E gene of SARS-CoV-2, both individually and simultaneously with sensitivity of down to 10 (singleplex RT-PCR) and 20 (multiplex RT-PCR) copies of RNA. Application in a quadruplex RT-PCR assay demonstrated simultaneous monitoring of all three SARS-CoV-2 genes from a complex mixture of RNA and successful detection of the RNase P transcript to ensure the integrity of the patient sample. Finally, direct comparison showed that Taq pol V2 and V3 can achieve a RT-PCR performance comparable to the two enzyme Luna^®^ Universal Probe One-Step RT-PCR Kit from NEB.

The one-enzyme formulation has several advantages over a two-enzyme based RT-PCR. Reaction parameters can be optimally adapted to a single DNA pol variant. This means that there is no need to find a compromise between the reaction conditions of two enzymes. Furthermore, the thermostability and the usage of the Taq pol aptamer enable a hot-start of the reaction minimizing the propensity for false amplification in case of high structured RNA^70^. Besides, RT-PCR does not necessarily require an extra RT step prior to amplification, which saves time. Moreover, there is no competition for the binding and thereby blocking of the RNA template, as may occur with two enzymes This aspect could potentially improve efficiency of detecting small amounts of the target molecules^42^. Since only 3 or 4 mutations need to be introduced into the wild-type Taq pol backbone, established production and purification processes can simply be retained, which saves current resources.

The mechanism by which these DNA pols acquire their properties is still unclear. All mutation sites are located within the polymerase domain, which likely allows for the proper binding of the DNA/RNA hybrid and/or the folding of the DNA pol into its active form after DNA/RNA binding^57,61^. It is known that amino acid N483 is located in the thumb domain of the Taq pol contacting the template strand^71^. Residues E507 and S515 are also part of the thumb domain and interact with the primer strand. Especially, the mutation E507K has been shown to contribute to a fast PCR cycling property of the Taq pol^72^. The amino acid I614 is located in the finger domain and forms part of the hydrophobic pocket that binds the nucleobase and deoxyribose of the incoming dNTP. Its mutation has been identified to affect the fidelity of nucleobase pairing^73^. Mutation I707L is placed in the finger domain and has been shown to reduce the polymerase activity at lower temperatures, while retaining normal activity at the optimal reaction temperature and thermostability at 95°C^69^. Overall, the mutation sites are distributed throughout the polymerase domain and might contribute to synergistic effects that are improving the properties of RT-PCR. Our results add new DNA pols to the toolbox of enzymes suitable to be used in RT-PCR methodologies and further demonstrate that DNA polymerase engineering is a viable strategy to obtain enzymes with suitable properties.

## Materials and methods

### Oligonucleotides

DNA oligonucleotides were ordered from IDT or Biomers in HPLC grade and directly used in RT-PCR assays and primer extension reactions. The RNA oligonucleotide was purchased PAGE purified from Purimex and was directly used. SARS-CoV-2 RNA was purchased from the European Commission Joint Research Centre (CRM code: EURM-019) and was directly used. The SARS-CoV-2 RNA was stored at −20 °C according to the manufacturer’s instructions and diluted samples were stored at −80 °C in aliquots. The universal human reference RNA was purchased from Invitrogen (catalogue number: QS0639) and was directly used. For primer extension reactions analysed by denaturing PAGE, oligonucleotides were purified by preparative PAGE prior to usage. Thereafter, radioactive labelling with [γ-^32^p]-ATP (Hartmann Analytic) and T4 polynucleotide kinase (NEB) was conducted following the manufacturer’s protocol. The concentration of the used oligonucleotides was determined by measuring the absorbance at 260 nm and applying the Lambert-Beer law. Used dNTPs were supplied by Jena Bioscience. DNA and RNA sequences of all oligonucleotides are shown in Supplementary Table S1.

### Taq pol library preparation

256 pGDR11 plasmids carrying different Taq pol genes were purchased from Genewiz (Supplementary Table S2) and delivered in one single tube. Plasmids were synthesized in equimolar amounts using the trimer-controlled gene synthesis approach (smart library) from Genewiz and quality checked by sequencing 96 individual clones. Transformation of electro competent *E. coli* BL21 DE3 (Stratagene, Supplementary Table S3) cells was performed by adding 10 ng plasmids to 100 µL cells and applying an electric pulse with 1800 V, 200 Ω and a pulse length of around 5 ms by Gene Pulser Xcell (Bio-Rad). Cells were mixed with 1 mL SOC medium and incubated at 37 °C for 1 h. After plating and incubation of the cells (37 °C, overnight), 100 µL of LB medium containing 100 µg/mL carbenicillin disodium salt was inoculated with a single cell colony to end up with 7 × 384 deep-well plates. Cells were grown at 37 °C overnight, 100 µL glycerol was added in each well and library plates were stored at −80 °C.

### Generation of *E. coli* cell lysates in 96 well plates

1 mL LB medium with 100 µg/mL carbenicillin disodium salt was inoculated with glycerol stocks of *E. coli* and cells were grown at 37 °C in a shaker. After reaching an OD_600_ of 0.6, expression was induced by the addition of IPTG (0.4 mM final concentration). Cells were incubated at 37 °C in a shaker for 3 h and then harvested by centrifugation with 4000 rpm at 4 °C for 30 min. Pellets were resuspended and lysed by the addition of 800 µL 1x lysis buffer I (50 mM Tris-HCl (pH 9.2), 16 mM (NH_4_)_2_SO_4_, 2.5 mM MgCl_2_, 0.1% (v/v) Tween 20, 0.5 mg/mL lysozyme) and incubation at 37 °C for 20 min. After denaturing of *E. coli* host proteins at 75 °C for 45 min, plates were centrifuged at 4000 rpm and 4 °C for 30 min and stored at 4 °C. Uniform expression was exemplary analysed by SDS-PAGE.

### Large scale protein production and purification

400 mL LB medium with 100 µg/mL carbenicillin disodium salt was inoculated with 4 mL of the respective *E. coli* overnight culture and incubated at 37 °C in a shaker. After reaching an OD_600_ of 0.6–0.8, induction was performed by adding 1 mM IPTG. Cells were incubated at 37 °C in a shaker for 4 h. Then, cultures were harvested by centrifugation at 4 °C and 4000 rpm for 30 min and pellets were stored at −20 °C. Cell pellets were resuspended and lysed in 10 mL 1x lysis buffer II (10 mM Tris-HCl (pH 9.2), 300 mM NaCl, 2.5 mM MgCl_2_, 0.1% (v/v) Triton X-100, 1 mg/mL lysozyme) at 37 °C for 20 min. After heat inactivating of *E. coli* host proteins at 75 °C for 45 min, lysates were ultra-centrifuged at 20.000 rpm for 1 h. 5 mM imidazole was added to the supernatant and metal ion-based affinity purification was conducted by applying the cOmplete™ His-Tag purification resin (Roche). Here, 3 mL beads were washed 4 times with 5 mL calibration buffer (10 mM Tris-HCl (pH 9.2), 300 mM NaCl, 2.5 mM MgCl_2_, 0.1% (v/v) Triton X-100, 5 mM imidazole) by alternate mixing and centrifugation at 1000 rpm and 4 °C for 3 min. The cell lysate was mixed with the calibrated beads and shaken at 4 °C in an overhead shaker overnight. The protein bead solution was applied onto a 15 mL chromatography column and the flow through was collected. Then, beads were washed 4 times with 5 mL washing buffer (10 mM Tris-HCl (pH 9.2), 300 mM NaCl, 2.5 mM MgCl_2_, 0.1% (v/v) Triton X-100, 20 mM imidazole). Protein elution was performed by loading 1.6 mL elution buffer I (100 mM Tris-HCl (pH 9.2), 5 mM MgCl_2_, 200 mM imidazole) 6 times and collecting the flow through separately at 4 °C. Protein purification was analysed by SDS-PAGE. Pure enzyme fractions were combined and concentrated by using an Amicon filter tube (Amicon Ultra-15, 30 kDa MWCO, Millipore) at 4 °C and 4000 rpm. Remaining imidazole was removed by washing the protein solution 4 times with 20 mL elution buffer II (100 mM Tris-HCl (pH 9.2), 5 mM MgCl_2_) followed by a final concentration step to obtain an end volume of 0.4–0.5 mL. For storage, 20x storage buffer (100 mM Tris-HCl (pH 9.2), 320 mM (NH_4_)_2_SO_4_, 5 mM MgCl_2_, 2% (v/v) Tween 20) was added in an amount equal to 1/9th of the volume of the protein (final 2x storage buffer). Finally, autoclaved glycerol was added to reach a final concentration of 50% and enzyme stocks were stored in aliquots at −20 °C. Protein concentration was determined by employing a Bradford assay (Roti^®^Quant, Roth) by using BSA as reference protein.

### Primer extension with radioactively labelled primer

Reactions were carried out in 10 µL end volume. The reaction mixture contained 225 nM RNA oligonucleotide template, 150 nM radioactively labelled DNA primer, 100 µM of dNTPs (each) and 2 nM KTq pol variant in 1x Taq reaction buffer (50 mM Tris-HCl (pH 9.2), 16 mM (NH_4_)_2_SO_4_, 2.5 mM MgCl_2_, 0.1% (v/v) Tween 20). First, template and primer were annealed by heating the mixture to 95 °C for 2 min and then cooling it down stepwise to 4 °C. Then, dNTPs in 1x Taq reaction buffer was added and pre-warmed to 55 °C. Primer extension was started by the addition of 4 µL KTq pol variant in 1x Taq reaction buffer. Reactions were allowed to proceed at 55 °C for indicated reaction times and were quenched by the addition of 20 µL stop solution (80% formamide, 20 mM EDTA, 0.25% (w/v) bromophenol blue, 0.25% (w/v) xylene cyanole). After denaturation at 95 °C for 5 min, reactions were analysed by 12% denaturing PAGE and visualised by phosphor imaging conducted on a Typhoon™ FLA 9500 (GE Healthcare Life Science). Reactions were repeated twice.

### RT-PCR assay using the RNA oligonucleotide as template

RT-PCRs were performed in a reaction volume of 10 µL containing 100 pM RNA oligonucleotide as template, 100 nM forward and reverse primer (each), 200 µM dNTPs (each), 0.5 M betaine, 1x SYBR green I, 100 nM Taq pol aptamer and 100 nM of Taq pol variants in 1x Taq reaction buffer (50 mM TrisHCl (pH 9.2), 16 mM (NH_4_)_2_SO_4_, 2.5 mM MgCl_2_, 0.1% (v/v) Tween 20). RT-PCR was conducted following the thermocycling protocol: 95 °C for 60 s, then 40 cycles of 95 °C for 15 s and 62 °C for 30 s. Fluorescent intensities were measured after each of the 40 cycles using the LightCycler^®^ 96 instrument (Roche) and the results were analysed using the Light Cycler^®^ 96 SW 1.1 software (Roche, absolute quantification mode). Reactions were performed two or three times.

### Screening assay for Taq pol variants with increased RT-PCR activity

For screening, 10 µL reaction mixture contained 250 µM dNTPs (each), 670 nM forward and reverse primer (each), 0.5 M betaine, 800 nM Taq pol aptamer, 3 µL of the respective *E. coli* cell lysate, 170 nM fluorescent probe and SARS-CoV-2 RNA template as indicated in 1x Taq reaction buffer (50 mM TrisHCl (pH 9.2), 16 mM (NH_4_)_2_SO_4_, 2.5 mM MgCl_2_, 0.1% (v/v) Tween 20). After preincubation at 61 °C for 150 s, 10 cycles with 95 °C for 5 s and 61 °C for 150 s were carried out. Then, 45 cycles with 95 °C for 10 s and 57 °C for 35 s followed. Fluorescent intensities were measured after each of the 45 cycles. PCR was monitored by using the CFX384 Touch Real-Time PCR Detection System (Biorad) and the Bio-Rad CFX Manager 3.1 software (single treshold mode). Reactions were performed in 384 well plates, thus 4 lysate plates (96 wells) could be analysed in parallel. For easy handling, pipetting was conducted by using the pipetting robot ASSIST plus from Integra. Enzyme performance was evaluated by assessing the Cq values of each Taq pol variant and the shape of the product curve. Product formation was exemplary analysed by agarose gel electrophoresis (2.5% agarose gel in 1x TAE buffer).

For reactions with purified Taq pol variants, 10 µL reaction mixture contained 250 µM dNTPs (each), 670 nM forward and reverse primer (each), 0.5 M betaine, 100 nM Taq pol aptamer, 30 nM respective Taq pol variant, 170 nM fluorescent probe or 1x SYBR green I and SARS-CoV-2 RNA template as indicated in 1x Taq reaction buffer (50 mM Tris-HCl (pH 9.2), 16 mM (NH_4_)_2_SO_4_, 2.5 mM MgCl_2_, 0.1% (v/v) Tween 20). After preincubation at 61 °C for 150 s, 10 cycles with 95 °C for 5 s and 61 °C for 150 s were carried out. Then, 35 cycles with 95 °C for 10 s and 57 °C for 35 s followed. Fluorescent intensities were measured after each of the 35 cycles. PCR was monitored by using the CFX384 Touch Real-Time PCR Detection System (Biorad) and the Bio-Rad CFX Manager 3.1 software (single treshold mode).

### Site directed mutagenesis for introducing I707L mutation

For PCR, Phusion High-Fidelity DNA Polymerase (NEB) was used according to the manufacturer’s protocol. The reaction mixture contained 200 µM dNTPs (each), 0.5 µM forward primer (which had the desired point mutation in its DNA-strand), 0.5 µM reverse primer (with a phosphate residue on its 5’-ending), 2 ng/µL plasmid DNA, 3% (v/v) DMSO (NEB) and 0.02 U/µL Phusion DNA Polymerase (NEB) in 1x Phusion HF buffer (NEB). The reaction had a total volume of 20 µL and was performed in triplicates. An initial denaturation step was performed at 98 °C for 30 s, followed by 30 PCR cycles. One cycle consisted of a denaturation step at 98 °C for 10 s, an annealing step at 60 °C for 15 s and an extension step at 72 °C for 160 s. Finally, the PCR was terminated by a final extension step at 72 °C for 5 min. To eliminate the methylated paternal DNA plasmid, a DpnI enzymatic digestion was conducted by adding 1 µL DpnI (NEB) and 5 µL CutSmart buffer (NEB, finally 1x) to 44 µL PCR reaction, resulting in a final volume of 50 µL. The digestion was carried out at 37 °C for 1.5 h. The product was purified by agarose gel electrophoresis (0.8% agarose gel in 1x TAE), excision of the respective bands and applying the NucleoSpin Gel and PCR clean-up kit (Macherey-Nagel). Finally, the mutated plasmid was ligated by adding 1 µL T4 DNA Ligase (NEB) and 2 µL T4 DNA ligase reaction buffer (NEB, finally 1x) to 17 µL purified DNA. Ligation was performed at 23 °C for 2 h and the enzyme was denatured at 65 °C for 10 min. The circular plasmid was transformed into electro competent *E. coli* BL 21 (DE3) using the same method as described for the Taq pol library preparation. After plating and incubation of the cells (37 °C, overnight), 6 mL of LB medium containing 100 µg/mL carbenicillin disodium salt was inoculated with a single cell colony. Cells were grown at 37 °C overnight and a glycerol stock was generated (500 µL cells mixed with 500 µL glycerol). The presence of the I707L mutation was verified by isolating and sequencing the plasmid.

### Singleplexed and multiplexed one-step RT-PCR using SARS-CoV-2 RNA as template

#### Singleplex RT-PCR

For the singleplex PCR, the CDC-approved DNA primers and fluorescent probes that target the SARS-CoV-2_N1, N2 and E genes were used in the reaction and are listed in Supplementary Table S1. 20 µL reaction mixture contained 250 µM dNTPs (each), 670 nM forward and reverse primer (each) addressing either the nCov_N1 or nCov_N2 gene, 0.5 M betaine, 0.5 mM MgCl_2_, 400 nM Taq pol aptamer, 120 nM respective Taq pol variant, 170 nM fluorescent probe addressing either the nCov_N1 or nCov_N2 gene and SARS-CoV-2 RNA template as indicated in 1x Taq reaction buffer (50 mM Tris-HCl (pH 9.2), 16 mM (NH_4_)_2_SO_4_, 2.5 mM MgCl_2_, 0.1% (v/v) Tween 20). When addressing the nCoV_E gene, the same reaction mix was used as described above, but 850 nM forward and reverse primer (each) and 216 nM fluorescent probe were applied. After preincubation at 65 °C for 150 s, 10 cycles with 95 °C for 5 s and 65 °C for 150 s were carried out. Then, 40 cycles with 95 °C for 10 s and 57 °C for 35 s followed. Fluorescent intensities were measured after each of the 40 cycles. The LightCycler^®^ 96 System (Roche) was used to monitor the reaction and the Light Cycler^®^ 96 SW 1.1 software (Roche, absolute quantification mode) was used to analyse the results. To determine the detection limits, the SARS-CoV-2 RNA template was diluted as indicated and applied in the singleplexed RT-PCR assay as explained above. Average Cq values were plotted against the decadic logarithm of the template concentrations and linear regression was employed. PCR efficiencies (E) were calculated using the slope (m) of the calculated linear function: $\text{E}={\text{10}}^{\text{(}\frac{\text{-1}}{\text{m}}\text{)}}-1$

Linear functions, coefficient of determination and PCR efficiencies are shown in Supplementary Table S4. Product formation was analysed by agarose gel electrophoresis (2.5% agarose gel in 1x TAE buffer). Reactions were performed at least twice.

#### Triplex RT-PCR

For the triplex PCR, the CDC-approved DNA primers and fluorescent probes that target the SARS-CoV-2_N1, N2 and E genes were used in the reaction and are listed in Supplementary Table S1. The same reaction volume and reaction mix was used as for the singleplex RT-PCR, but all primer/probe sets targeting the nCov_N1, N2 and E gene were simultaneously added in the same concentrations as above. The same thermocycling protocol was conducted as for the singleplex RT-PCR above. The LightCycler^®^ 96 System (Roche) was used to monitor the reaction and the Light Cycler^®^ 96 SW 1.1 software (Roche, absolute quantification mode) was used to analyse the results. To determine the detection limits, the SARS-CoV-2 RNA template was diluted as indicated and applied in the multiplexed RT-PCR assay. Average Cq values were plotted against the decadic logarithm of the template concentrations and linear regression was employed. PCR efficiencies (E) were calculated as stated above. Linear functions, coefficient of determination and PCR efficiencies are shown in Supplementary Table S4. Product formation was analysed by agarose gel electrophoresis (2.5% agarose gel in 1x TAE buffer). Reactions were performed at least twice.

#### Quadruplex RT-PCR

For the quadruplex PCR, the CDC-approved DNA primers and fluorescent probes targeting the SARS-CoV-2_N1, N2 and E genes and the RNase P mRNA were used in the reaction (Supplementary Table S1). The same reaction volume and reaction mix was used as for the singleplex RT-PCR, but all primer/probe sets targeting the nCov_N1, N2 and E gene were simultaneously added in the same concentrations as before. Additionally, 670 nM forward and reverse primer (each), 170 nM fluorescent probe for the RNase P detection and 1 ng/µL universal human reference RNA were added. The same thermocycling protocol was conducted as for the singleplex RT-PCR above. The LightCycler^®^ 96 System (Roche) was used to monitor the reaction and the Light Cycler^®^ 96 SW 1.1 software (Roche, absolute quantification mode) was used to analyse the results. Furthermore, The SARS-CoV-2 RNA template was diluted as indicated and applied in the multiplexed RT-PCR assay. Average Cq values were plotted against the decadic logarithm of the template concentrations and linear regression was employed. PCR efficiencies (E) were calculated as stated above. Linear functions, coefficient of determination and PCR efficiencies are shown in Supplementary Table S4. Product formation was analysed by agarose gel electrophoresis (2.5% agarose gel in 1x TAE buffer). Reactions were performed at least twice.

#### Quadruplex RT-PCR with Luna® Universal probe one-step RT-PCR kit

For the quadruplex PCR, the CDC-approved DNA primers and fluorescent probes targeting the SARS-CoV-2_N1, N2 and E genes, as well as the RNase P mRNA, were used in the reaction (Supplementary Table S1). The reaction was optimized according to the manufacturer’s recommendations. 20 µL reaction mixture contained 670 nM of each forward and reverse primer and 170 nM of each fluorescent probe for the nCov_N1 and N2 genes, as well as 850 nM of each forward and reverse primer and 216 nM of fluorescent probe for the nCov_E gene. Additionally, 670 nM of each forward and reverse primer and 170 nM fluorescent probe were included for RNase P detection. The reaction was carried out with 1 ng/µL universal human reference RNA, 10.000 c/µL of the SARS-CoV-2 RNA template and 1x Luna WarmStart RT enzyme mix in 1x Universal Probe One-Step Reaction Mix (NEB). When using the engineered Taq pol variants, the Luna WarmStart RT enzyme mix was replaced with 120 nM of the respective Taq pol variant and 400 nM Taq pol aptamer. One-step RT-PCR was carried out using the following cycling protocol: After preincubation at 60 °C for 10 min, an initial denaturation step at 95 °C for 1 min was performed, followed by 45 cycles of 95 °C for 10 s and 60 °C for 1 min. Fluorescent intensities were measured after each of the 45 cycles. The LightCycler^®^ 96 System (Roche) was used to monitor the reaction and the Light Cycler^®^ 96 SW 1.1 software (Roche, absolute quantification mode) was used to analyse the results.

### DNA amplicon library preparation for NGS analysis

RT-PCR with Taq pol variants was conducted similarly to the RT-PCR screening approach using purified enzymes. 10 µL reaction mix contained 250 µM dNTPs (each), 670 nM forward and reverse primer (each, giving the 100 bp transcript from the nCov_N1 gene, Supplementary Table S1), 0.5 M betaine, 100 nM Taq pol aptamer, 30 nM respective Taq pol variant, 10.000 copies of SARS-CoV-2 RNA template in 1x Taq reaction buffer (50 mM Tris-HCl (pH 9.2), 16 mM (NH_4_)_2_SO_4_, 2.5 mM MgCl_2_, 0.1% (v/v) Tween 20). After preincubation at 61 °C for 150 s, 10 cycles with 95 °C for 5 s and 61 °C for 150 s were carried out. Then, 35 cycles with 95 °C for 10 s and 57 °C for 35 s followed.

To determine the DNA product concentration, each reaction mix was analysed by using the 4150 TapeStation System (Agilent), High Sensitivity D1000 ScreenTapes (Agilent) and High Sensitivity D1000 Reagents (Agilent). Sample preparation and measurement was carried out according to the guidelines of the company. Each reaction was loaded twice and 1 ng of a 100 bp reference DNA with the same sequence was applied as a control. Afterwards, remaining reaction mix was purified by agarose gel electrophoresis (2.5% agarose gel in 1x TAE buffer), excision of the respective band and applying the NucleoSpin Gel and PCR clean-up kit XS (Macherey-Nagel). Elution was achieved by adding 7 µL elution buffer to the column (for better recovery, elution was loaded again). DNA concentration was determined by using the 4150 TapeStation System (Agilent) as described above. In the next step, DNA was repaired by using 0.35 nM purified DNA, 0.1 µL PreCR Repair mix (NEB) with 100 µM of dNTP (each), 1x NAD^+^ (provided by the manufacturer) and 1x ThermoPol reaction buffer (NEB) to reach 20 µL reaction volume. Repair was performed at 37 °C for 20 min. Unique molecular identifier (UMI) introduction was obtained by applying Q5 Hot Start High-Fidelity DNA Polymerase (NEB) in a three-step PCR reaction. The reaction mixture contained 20 µL repair mix, 0.02 U/µL DNA polymerase, 200 nM forward and reverse primer (each) and finally 0.2x Q5 reaction buffer to reach 25 µL reaction volume. After initial denaturation at 98 °C for 2 min, two cycles with 98 °C for 10 s, 62 °C for 30 s and 72 °C for 30 s were carried out followed by a final elongation step at 72 °C for 2 min. Reaction was purified by agarose gel electrophoresis as described above. This time, buffer NTI was diluted with Milli-Q water in a ratio of 2:1 and then used for dissolving gel pieces at 50 °C. DNA concentration was determined with a qPCR approach, where an DNA oligonucleotide with a similar length was applied as reference in different template concentrations (200 pM–0.01 pM). 1x NEBNext Ultra II Q5 Mastermix (NEB) was used for PCR with 1.6 µL DNA, 400 nM forward and reverse primer (each), 1x SYBR green I in 5 µL reaction volume. After a final denaturation step at 98 °C for 1 min, a three-step cycling protocol with 35 PCR cycles followed with 98 °C for 10 s, 60 °C for 30 s and 72 °C for 30 s and a final elongation step with 72 °C for 2 min was conducted. Fluorescent intensity was measured after each of the 35 cycles (LightCycler^®^ 96 System (Roche)) and melting curves were determined at the end of the reaction. The Cq values were determined using the Light Cycler^®^ 96 SW 1.1 software. For analysis, Cq values of reactions with reference DNA were plotted against the decadic logarithm of the template concentrations and linear regression was employed. DNA concentrations of samples to be analysed were calculated using the linear predictor function. Results were multiplied by two. DNA was repaired once again by using 0.05 µL PreCR mix (NEB), 1x NAD^+^, 100 µM of dNTP (each), 48.9 fM DNA and 1x ThermoPol reaction buffer (NEB) to reach 11.25 µL end volume. After reaction for 20 min at 37 °C, DNA mix was directly used for Amplicon PCR. Here, 400 nM forward and reverse primer (equipped with Illumina adapter sequence and indices), the entire repair mix (final DNA concentration 22 fM) and 1x NEBNext Ultra II Q5 Mastermix (NEB) was used for reaction in 25 µL end volume. After an initial denaturation step at 98 °C for 5 min, a three-step cycling protocol with 35 PCR cycles followed with 98 °C for 10 s, 70 °C for 30 s and 72 °C for 30 s and a final elongation step at 72 °C for 2 min was conducted. Reaction was analysed and purified by agarose gel electrophoresis as described above. To dissolve gel pieces, buffer NTI (Macherey Nagel) was diluted with Milli-Q water in ration 2:1 and 450 µL NTI mix was added to the gel pieces. After complete dissolving at 50 °C, the mixture was diluted once again with MilliQ water to reach a final volume of 1750 µL. DNA was loaded on the column and eluted with 20 µL elution buffer (load twice). After performing the repair reaction on the whole eluate as described above (25 µL reaction volume), DNA was purified by using the QIAEX II System (Qiagen). Here, 2 µL particle slurry was used for each sample and elution of DNA was achieved by adding 18 µL Milli-Q water to the dried beads, incubation for 5 min and taking 16 µL after centrifugation. The concentration of the final DNA library was determined by Quantus™ Fluorometer (Promega) and ranged between 19 and 64 nM. After quality control with Bioanalyzer 2100 (Agilent), DNA libraries were pooled and sequenced in paired-end mode (2*75 bp) on an IIlumina NextSeq2000 instrument. For RT-Taq, Mut_RT and Taq pol V2, library preparation was performed twice. Each DNA library was sequenced once and the resulting coverage was used for error calculation (Supplementary Table S5).

### NGS data processing of amplicon libraries

Sequencing data were processed by using the software KNIME (Supplementary Table S6). Quality and sequence are read from the FastQ files. Phred quality scores (Q scores) were transformed into base calling error probabilities (P) by using: $\text{P}={\text{10}\text{}}^{\frac{\text{-Q}}{\text{10}}}$

Read1 and Read 2 were merged giving the expected size of transcript. The highest quality data was used in the overlapping regions of Read1 and Read2. High quality data were filtered and aligned to the RNA reference of the SARS-CoV-2 RNA sequences. A frameshift correction was performed e.g. caused by phasing or pre-phasing during sequencing^74,75^. Those reads with a misalignment higher than 24% were removed from the data set (removal of only 0.5% of the reads such as adapter dimers). Reads were sorted into UMI families and family read numbers were counted. Substitution error calculation was performed in each UMI family having more than 3 reads. If 90% of the reads within each family carry the misincorporation, the error is set to 1 (otherwise 0). Afterwards, the substitution error was calculated over all UMI families at each position of the transcript. To normalize the error rates of each Taq pol variant according to its amplification activity, the individual amplification and doubling numbers of each enzyme were included in the calculation. The amplification number (A) and the doubling number (D) were determined by using the following equations (n = amount of substance [mol]):

$$\text{A}=\text{}\frac{\text{n(after PCR)}}{\text{n(input RNA)}}\qquad 2^\text{D}=\text{A}\rightarrow\text{D}=\frac{\text{Ln(A)}}{\text{Ln(2)}}$$

Each error rate for each position in the template was divided by the individual doubling number and plotted using Microsoft excel software. For the average substitution error, the region of the amplicon without primer binding sites is included (area depicted in Supplementary Fig. S5). The last T read in this region was excluded since values on this position differ very clearly from the rest of the data. The coverage, the number of UMI families and the doubling numbers used for error calculation are listed individually for each sequencing library in Supplementary Table S5.

## Electronic supplementary material

Below is the link to the electronic supplementary material.

Supplementary Material 1

## Data Availability

HTS data are available in the NCBI GEO database, records GSE280134, (https://www.ncbi.nlm.nih.gov/geo/query/acc.cgi?acc=GSE280134, private secure token: ibytqmygljivrkz). All data needed to evaluate the conclusions in the paper are present in the paper and/or in the Supplementary data. Crystal structure information is available at the RCSB PDB Protein Data Bank under the accession number 4BWM (https://www.rcsb.org/structure/4BWM) and 1TAQ (https://www.rcsb.org/structure/1TAQ). Additional data may be requested from the authors (A) M. and L. (B) H.
