# Supplementary material for: Engineering of novel DNA polymerase variants for single enzyme quantitative multiplex reverse transcription-PCR
Source: Sci Rep. 2025 Jul 18;15:26147. doi: 10.1038/s41598-025-10211-x (PMC12274487; doi:10.1038/s41598-025-10211-x)
Supplement: Supplementary file 1 — Supplementary Material 1 [file 41598_2025_10211_MOESM1_ESM.pdf]

Supplementary Data

Engineering of novel DNA polymerase variants for single enzyme quantitative multiplex Reverse Transcription-PCR

Authors: Luisa B. Huber[1], Virginie Marchand[2], Melike Somtürk[3], Silke Müller[4] and Andreas Marx[1,*]

Supplementary Tables

Table S1: RNA/DNA oligonucleotides used in this study.

**Preliminary experiments using parental DNA polymerases**	
Oligonucleotide	Sequence
Artificial RNA oligonucleotide	5'-AUAGGGGAAUGGGCCGUUCAUCUGCUAAAAGGUCUGCUUUUGGGGCUUGUAGU-3'
DNA primer rev	5'-ACTACAAGCCCCAAAAGCAG-3' ‡
DNA primer fwd	5'-ATAGGGGAATGGGCCGTTC-3'
Taq pol aptamer	5'-CGATCATCTCAGAACATTCTTAGCGTTTTGTTCTTG TGTATGATCG-3'
**Screening for RT-PCR active DNA polymerases**	
SARS-CoV-2 RNA European Commission Joint Research Centre (EURM-019)	5'-GGGAGACGAAUUGGGCCCUCUAGAUGCAUGCUCGAGCGGCCGCCAGUGUGAUGGAUAUCU<br>GCAGAAUUCGCCCUUAUUCAAGUAUUGAGUGAAAUGGUCAUGUGUGGCGGUUCACUAUAUGUU<br>AAACCAGGUGGAACCUCAUCAGGAGAUGCCACAACUGCUUAUGCUAAUAGUGUUUUUAACAUU<br>UGUCAAGCUGUCCGGAAGAGACAGGUACGUUAAUAGUUAAUAGCGUACUUCUUUUUCUUGCUU<br>UCGUGGUAUUCUUGCUAGUUACACUAGCCAUCCUUACUGCGCUUCGAUUGUGUGCGUACUGC<br>UGCAAUAUUGUUAACGUAUAAUGGACCCCAAAAUCAGCGAAAUGCACCCCGCAUUACGUUUGG<br>UGGACCCUCAGAUUCAACUGGCAGUAACCAGAAUGGAGAACGCAUUGCAACUGAGGGAGCCUU<br>GAAUACACCAAAAGAUCACAUUGGCACCCGCAAUCCUGCUAACAAUGCUGCAAUCGUGCUACAA<br>CUUCCUCAAGGAAAUUUUGGGGACCAGGAACUAAUCAGACAAGGAACUGAUUACAAACAUUGG<br>CCGCAAAUUGCACAAUUUGCCCCCAGCGCUUCAGCGUUCUUCGGAAUGUCGCGCAUUGGCAU<br>GGAAGUCACACCUUCGGGAACGUGGUUGACCUACACAGGUGCCAUCAAAUUGGAGUGUGACAU<br>ACCCAUUGGUGCAGGUAUAUGCGCUAGUUAUCAGACUCAGACUAAUUCUCCUCGGCGGGCAC<br>GUAGUGUAGCUAGUCAACCUGCUUUUGCUCGCUUGGAUCCGAAUUCAAAGGUGAAAUUGUUAU<br>CCGCUCACAAUUCCACACAACAUACGAGCCGGAAGCAUAAAGUGUAAAGCCUGGGGUGCCUAA<br>UGA-3'
DNA primer rev 1	5'-TCTGGTTACTGCCAGTTGAATCTG-3'
DNA primer 72 bp fwd 1	5'-GACCCCAAAATCAGCGAAAT-3'
DNA primer 100 bp fwd 2	5'-ACTGCTGCAATATTGTTAACGTAT-3'
DNA primer 156 bp fwd 3	5'-CGTGGTATTCTTGCTAGTTACAC-3'
DNA primer 208 bp fwd 4	5'-CGGAAGAGACAGGTACGTTA-3'
DNA primer 254 bp fwd 5	5'-CACAACTGCTTATGCTAATAGTGT-3'
TaqMan probe 1	5'-(FAM)-ACCCCGCATTACGTTTGGTGGACC-(BHQ1)-3'
Sanger Sequencing primer 1	5'-GAATGGACCGAAGAAGCAG-3'
Sanger Sequencing primer 2	5'-GTTCTGAGGTCATTACTGG-3'
**Introducing I707L mutation**	
Mutation primer fwd	5'-AGTTCGTGCCTGGCTTG-3'
Mutation primer rev	5'-(P)-TTCGGAAAGCTCTGAAAATAACG-3'
**Multiplex RT-PCR**	
nCov_N1 CDC fwd primer	5'-GACCCCAAAATCAGCGAAAT-3'
nCov_N1 CDC rev primer	5'-TCTGGTTACTGCCAGTTGAATCTG-3'
nCov_N1 CDC probe	5'-(FAM)-ACCCCGCATTACGTTTGGTGGACC-(BHQ1)-3'
nCov_N2 CDC fwd primer	5'-TTACAAACATTGGCCGCAAA-3'
nCov_N2 CDC rev primer	5'-GCGCGACATTCCGAAGAA-3'
nCov_N2 CDC probe	5'-(Sun554)-ACAATTTGC-ZEN-CCCCAGCGCTTCAG-(Iowa Black FQ)-3'
nCov_E CDC fwd primer	5'-ACAGGTACGTTAATAGTTAATAGCGT-3'
nCov_E CDC rev primer	5'-ATATTGCAGCAGTACGCACACA-3'
nCov_E CDC probe	5'-(Texas Red 617)-ACACTAGCCATCCTTACTGCGCTTCG-(Iowa Black RQ)-3'
RNase P CDC fwd primer	5'-AGATTTGGACCTGCGAGCG-3'
RNase P CDC rev primer	5'-GAGCGGCTGTCTCCACAAGT-3'
RNase P CDC probe	5'-(Cy5 668)-TTCTGACCT-ZEN-GAAGGCTCTGCGCG-(BHQ2)-3'

Universal Human Reference RNA	Invitrogen™ catalogue number: QS0639
**DNA library preparation for NGS**	
RT-PCR fwd primer	5'-ACTGCTGCAATATTGTTAACGTAT-3'
RT-PCR rev primer	5'-TCTGGTTACTGCCAGTTGAATCTG-3'
DNA primer UMI fwd 1	5'-GGAGTTCAGACGTGTGCTCTTCCGATCTNNNNNNNNACTGCTGCAATATTGTTAACGTAT-3' *
DNA primer UMI rev 1	5'-CTTTCCCTACACGACGCTCTTCCGATCTNNNNNNNNTATCTGGTTACTGCCAGTTGAATCTG-3' *
DNA primer UMI fwd 2	5'-GGAGTTCAGACGTGTGCTCTTCCGATCTNNNNNNNNCACTGCTGCAATATTGTTAACGTAT-3' *
DNA primer UMI rev 2	5'-CTTTCCCTACACGACGCTCTTCCGATCTNNNNNNNNATCTGGTTACTGCCAGTTGAATCTG-3' *
DNA primer UMI fwd 3	5'-GGAGTTCAGACGTGTGCTCTTCCGATCTNNNNNNNNGCACTGCTGCAATATTGTTAACGTAT-3' *
DNA primer UMI rev 3	5'-CTTTCCCTACACGACGCTCTTCCGATCTNNNNNNNNTCTGGTTACTGCCAGTTGAATCTG-3' *
DNA primer rev qPCR assay	5'-CTTTCCCTACACGACGCTCTTCCGAT-3'
DNA primer fwd qPCR assay	5'-GGAGTTCAGACGTGTGCTCTTCCGAT-3'
DNA primer 1 rev Amplicon	5'-AATGATACGGCGACCACCGAGATCTACACTATAGCCTACACTCTTTCCCTACACGACGCTCTTCCGATCT-3'
DNA primer 2 rev Amplicon	5'-AATGATACGGCGACCACCGAGATCTACACATAGAGGCACACTCTTTCCCTACACGACGCTCTTCCGATCT-3'
DNA primer 1 fwd Amplicon	5'-CAAGCAGAAGACGGCATACGAGATCGAGTAATGTGACTGGAGTTCAGACGTGTGCTCTTCCGATC-3'
DNA primer 2 fwd Amplicon	5'-CAAGCAGAAGACGGCATACGAGATTCTCCGGAGTGACTGGAGTTCAGACGTGTGCTCTTCCGATC-3'
DNA primer 3 fwd Amplicon	5'-CAAGCAGAAGACGGCATACGAGATAATGAGCGGTGACTGGAGTTCAGACGTGTGCTCTTCCGATC-3'

‡5'-radioactively labelled in case of primer extension experiments
*Nucleotides marked as "N" are random nucleotides used as indices (unique molecular identifier = UMI)

Table S2: Plasmids used in this study.

Designation	vector	description	Antibiotic resistance	Source
pTaq DNA pol	pGDR11	Gene of Taq pol variants, N-terminal 6xHis-tag, T5 promotor/lac operator	Ampicillin/Carbenicillin	derivative of pQE31 from Qiagen

Table S3: *E. coli* strains used in this study.

Designation	Genotype	Source
E. coli BL21 (DE3)	*B F⁻ ompT dcm lon hsdSB(r_B^- m_B^-) gal λ(DE3 [lacI lacUV5-T7 gene 1 ind1 sam7 nin5]) [malB+]K-12(λS)*	Stratagene

Table S4: Linear function, R^2 values and PCR efficiencies for singleplex and multiplex RT-PCR.

RT-PCR	Taq pol variant	Target gene	Linear function	R^2	PCR efficiency
Singleplex (Fig. 5 C)	RT-Taq	nCov_N1	-	-	-
		nCov_N2	y = -3.565x+25.08	0.9988	91%
		nCov_E	-	-	-
	Taq pol V2	nCov_N1	y = -3.514x+28.39	0.9984	93%
		nCov_N2	y = -3.487x+26.82	0.9974	94%
		nCov_E	y = -5.055x+40.92	1.00	58%
	Taq pol V3	nCov_N1	y = -3.587x+28.54	0.9995	90%
		nCov_N2	y = -3.559x+26.84	0.9981	91%
		nCov_E	y = -4.346x+32.87	0.9998	70%
	Taq pol V2 IL	nCov_N1	y = -3.508x+28.99	0.9968	93%
		nCov_N2	y = -3.489x+29.13	0.9914	93%
		nCov_E	y = -4.330x+33.30	0.9950	70%
triplex (Fig. 5 D)	RT-Taq	nCov_N1	-	-	-
		nCov_N2	y = -3.233x+24.15	0.9854	104%
		nCov_E	-	-	-

	Taq pol V2	nCov_N1	y = -3.375x+27.68	0.9997	98%
		nCov_N2	y = -3.453x+26.13	0.9956	95%
		nCov_E	y = -5.580x+36.72	1.00	51%
	Taq pol V3	nCov_N1	y = -3.458x+28.07	0.9997	95%
		nCov_N2	y = -3.317x+26.37	0.9957	100%
		nCov_E	y = -4.170x+30.86	0.9915	74%
	Taq pol V2 IL	nCov_N1	y = -3.436x+28.84	0.9996	95%
		nCov_N2	y = -3.530x+28.81	0.9974	92%
		nCov_E	y = -3.695x+30.54	0.9996	86%
quadruplex (Fig. 6 B)	RT-Taq	nCov_N1	-	-	-
		nCov_N2	y = -0.250x+13.24	0.05028	-
		nCov_E	-	-	-
		RNase P	y = -0.153x+13.24	0.2031	-
	Taq pol V2	nCov_N1	y = -3.340x+26.80	0.9445	99%
		nCov_N2	y = -3.426x+26.39	0.9445	96%
		nCov_E	y = -7.000x+44.32	0.9922	36%
		RNase P	y = -0.545x+15.60	0.4072	-
	Taq pol V3	nCov_N1	y = -3.233x+28.07	0.9504	104%
		nCov_N2	y = -3.421x+27.44	0.9189	96%
		nCov_E	y = -3.509x+28.18	0.9351	93%
		RNase P	y = -0.313x+14.92	0.2619	-
	Taq pol V2 IL	nCov_N1	y = -3.762x+28.69	0.9291	84%
		nCov_N2	y = -4.166x+30.39	0.8851	74%
		nCov_E	y = -4139x+30.54	0.9091	74%
		RNase P	y = -0.624x+16.33	0.2381	-

Table S5: Coverage for various NGS libraries generated by RT-PCR from SARS-CoV-2 RNA catalyzed by different Taq pol variants. The listed coverage values and the numbers of unique molecular identifier (UMI) families were included in the error calculation.

Figure	enzyme	doubling number	RNA	Coverage	UMI families
Supplementary Figure S5	RT-Taq	23.6	*100 nt section of SARS-CoV-2 N1 gene*	5118018	140782
	Mut_RT	22.5		5932086	143775
	Taq pol V2	27.0		6087315	131738
	Taq pol V3	27.1		6192132	134570
	Taq pol V2 IL	27.0		5507397	123795

Table S6: Software used in this study.

Software	Version	Reference
Image lab	6.1	https://www.bio-rad.com/de-de/product/image-lab-software?ID=KRE6P5E8Z
Roche LightCycler® 96 SW	1.1	https://www.roche.de/diagnostik/produkte-loesungen/systeme/lightcycler-systeme
Bio-Rad CFX Manager	3.1	https://www.bio-rad.com/de-de/sku/1845000-cfx-manager-software?ID=1845000
TapeStation Software	4.1.1	https://www.agilent.com/en/product/automated-electrophoresis/tapestation-systems/tapestation-software/tapestation-software-379381
KNIME	4.6.2	https://www.knime.com/
The PyMOL Molecular Graphics System	2.4	https://pymol.org/2/
GraphPad Prism	6.0	www.graphpad.com

SUPPLEMENTARY FIGURES

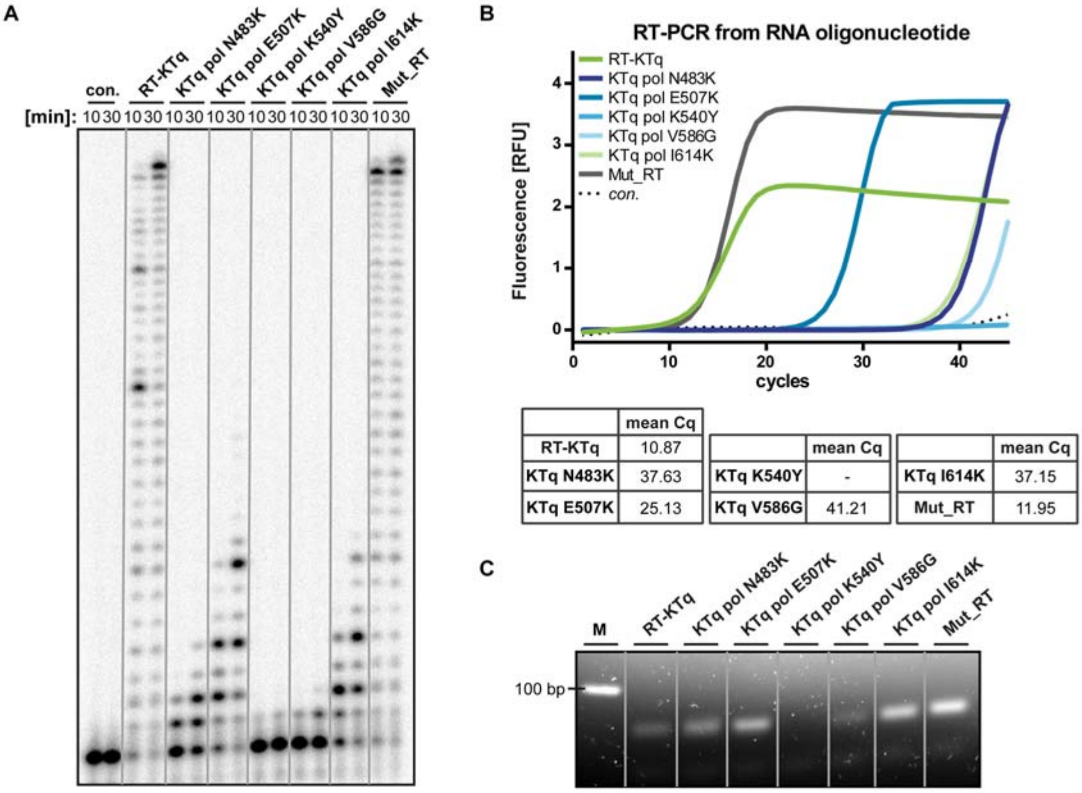

	mean Cq		mean Cq		mean Cq
RT-KTq	10.87				
KTq N483K	37.63	KTq K540Y	-	KTq I614K	37.15
KTq E507K	25.13	KTq V586G	41.21	Mut_RT	11.95

Figure S1: Comparison of RT-KTq, Mut_RT and all single mutants of Mut_RT regarding linear RT- and PCR activity. A) Primer extension with radioactively labelled primer from the artificial RNA oligonucleotide. Reaction mix contained 2 nM KTq pol variant (as indicated), 200 µM dNTPs (each), 150 nM labelled primer and 225 nM RNA template. Reaction was conducted for 10 and 30 min. Control lane (con.) depicts the signal for the primer only. Analysis was performed by 12% denaturing PAGE and phosphor imaging (Thyphoon FLA 9000 (GE Healthcare) & Image Lab 6.1 (BioRAD)). Original phosphor imaging scan is depicted in Figure S8. B) Amplification curves after RT-PCR from the artificial RNA oligonucleotide. Reaction mix contained 100 pM template, 100 nM forward and reverse primer, 200 µM dNTPs (each), 100 nM KTq pol variant (as indicated) and 1x SYBR green I. The control Mix (con.) contained the same mastermix, but the amount of RNA template was replaced by water (Cq of the NTC = n/a). RT-PCR were performed in duplicates. The mean of Cq values are depicted in the table. C) Analysis of product formation by agarose gel electrophoresis (2.5% gel). Gel Doc XR+ Gel Documentation System (BioRad) and Image Lab 6.1 (BioRad) was used for imaging. The uncropped agarose gel is found in Figure S9 A.

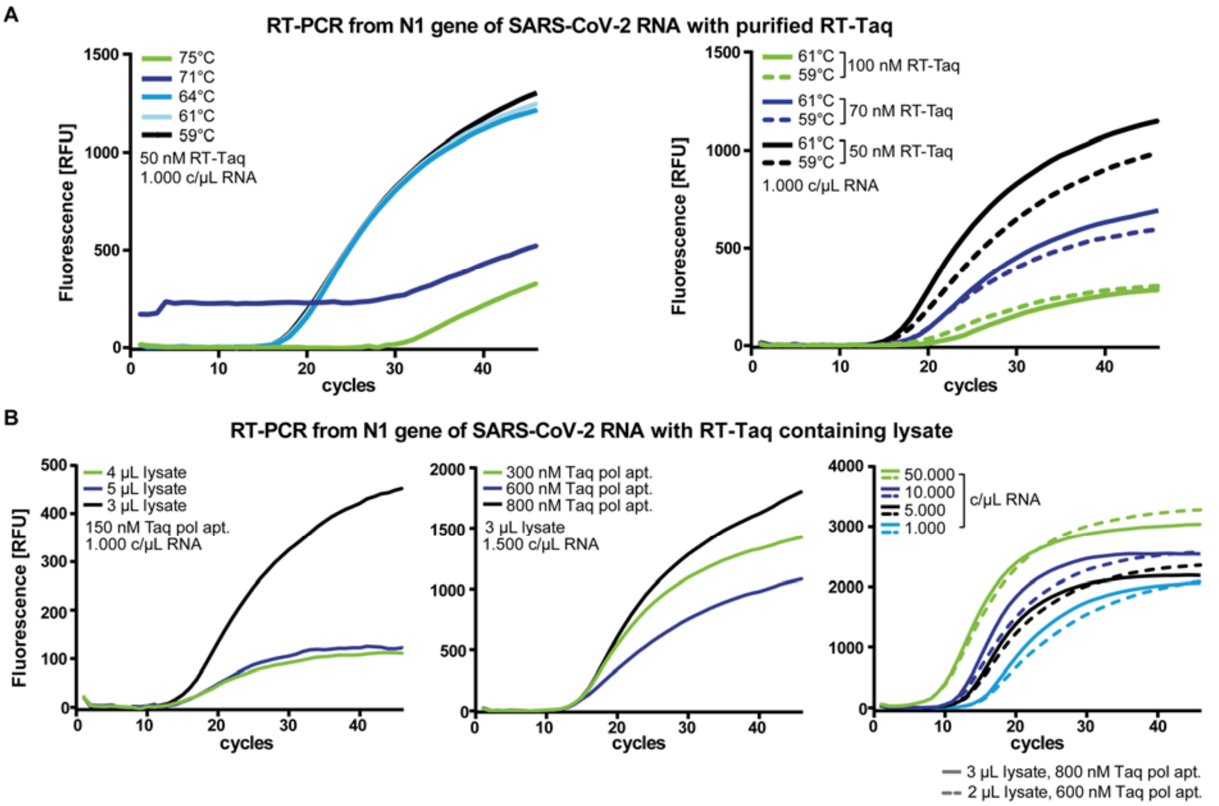

Figure S2: Determination of suitable screening conditions by conducting RT-PCR from SARS-CoV-2 RNA with RT-Taq. A) Purified RT-Taq (concentrations as indicated), 150 nM Taq pol aptamer and 1000 c/µl RNA was present in the reaction mix. A temperature gradient was used to find the suitable annealing temperature of 61°C. B) RT-PCR was catalysed by cell lysates containing the RT-Taq enzyme (lysate volume as indicated). Left: Appropriate lysate volume was determined to be 3 µL, middle: 800 nM Taq pol aptamer was determined to be suitable for screening, and left: 1000 c/µL RNA was found to be sufficient to monitor the reaction as the amount of RNA template has to be kept low in view of the size of the screening. The reactions were performed only once.

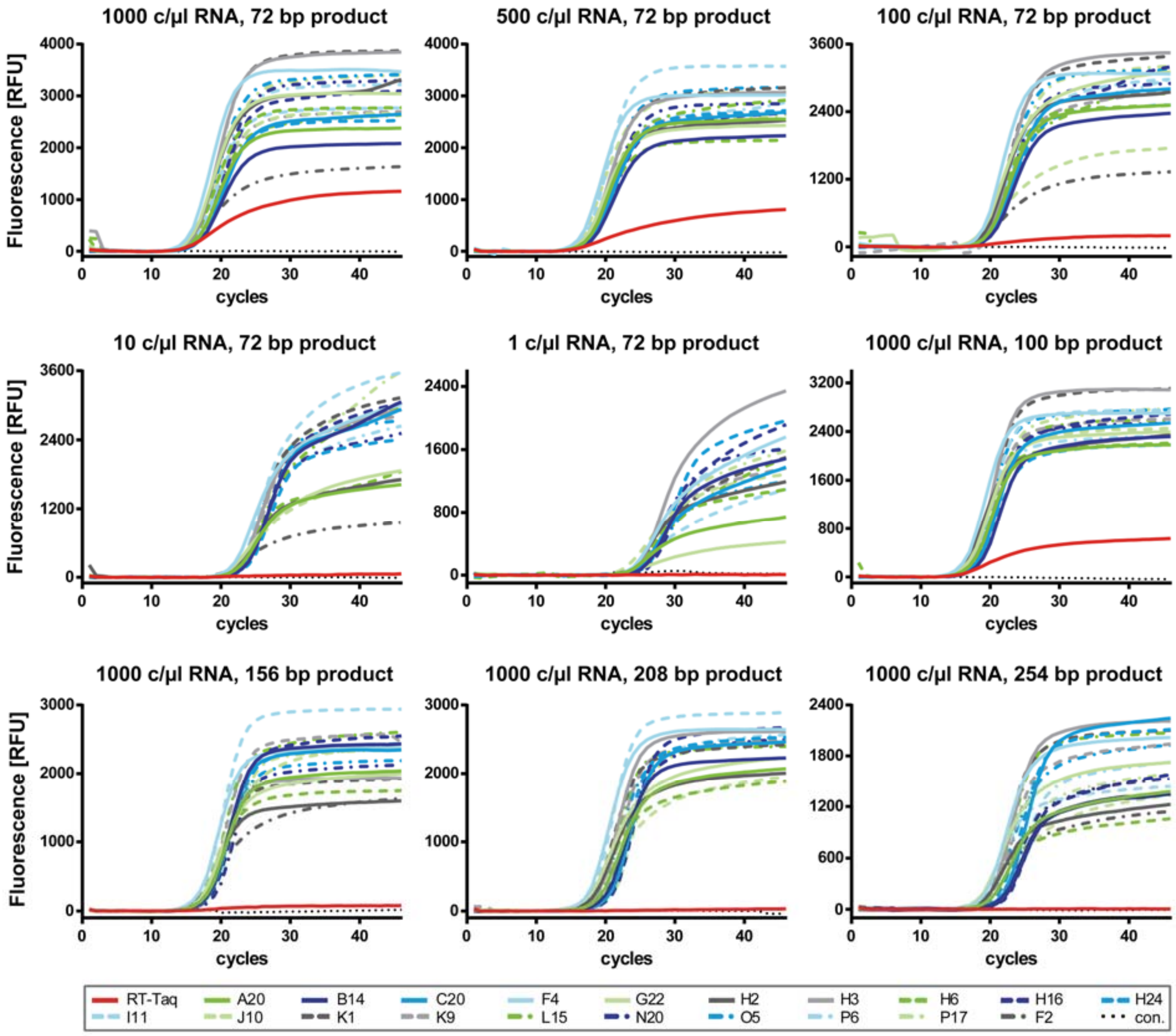

Figure S3: Amplification curves after RT-PCR from SARS-CoV-2 RNA catalysed by 20 best performing Taq pol lysates. 3 µl lysate, 800 nM Taq pol aptamer, 670 nM primer, 170 nM TaqMan probe and SARS-CoV-2 RNA (concentrations as indicated) were present in the reaction mix. Fwd primer was varied to generate PCR products with different lengths (as indicated). The same mastermix was used for the control reaction (con.), but the amount of RNA template was replaced by water. Of note, lysate F2 was only tested for 5 conditions. The reactions with 1000 c/µL template and 72 bp amplicon size were performed twice and other experiments were conducted only once.

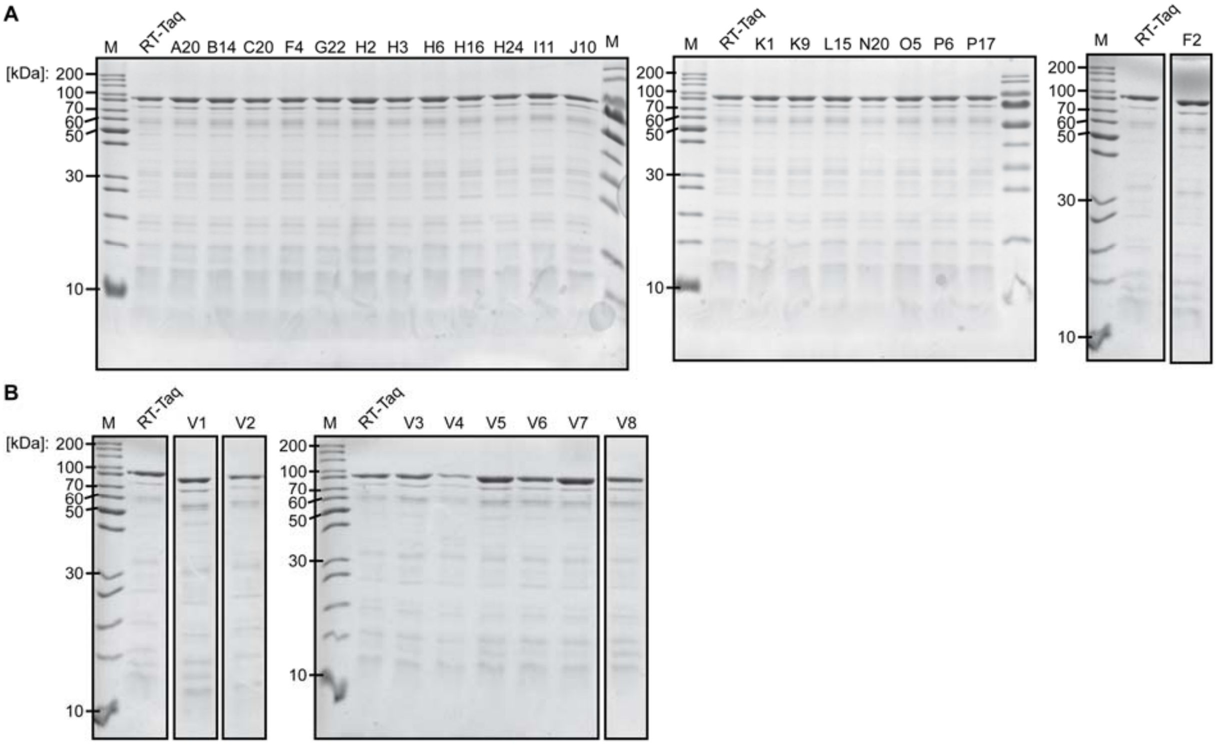

Figure S4: SDS-PAGE analysis of cell lysates containing Taq pol variants. Amersham Imager 600 RGB (Ge Healthcare Life Sciences) and Image Lab 6.1 (BioRad) was used for imaging. A) Lysates from the 20 most promising variants were analysed. Lysate bands on the right sight (RT-Taq and F2) were loaded on the same gel. Uncropped gel images are indicated in Figure S9 B, C, Figure S10 A. B) Lysates from the inactive variants were analysed. Lysate bands on the left site (RT-Taq, V1 and V2) were loaded on the same gel. Lysate bands on the right site (RT-Taq, V3–V7, V8) were loaded on the same gel. Uncropped gel images are indicated in Figure S10.

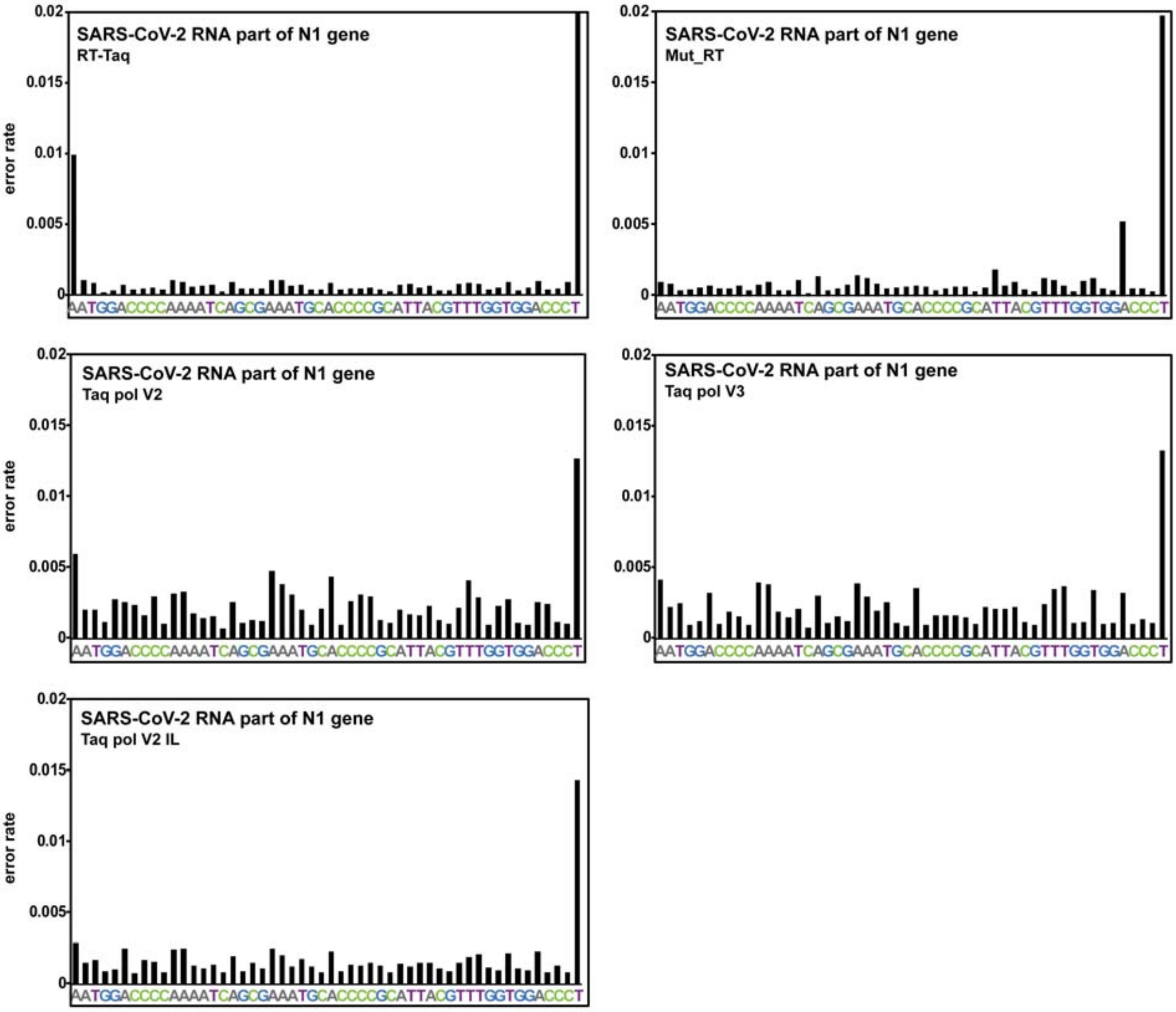

Figure S5: Investigation of the polymerase fidelity by NGS. Error rates from RT-Taq, Mut_RT, Taq pol V2, V3, V2 IL processing the SARS-CoV-2 RNA in the N1 region are illustrated (shown is the area of the amplicon without primer binding sites). 30 nM Taq pol variant and 100 nM Taq pol

aptamer was used for RT-PCR.

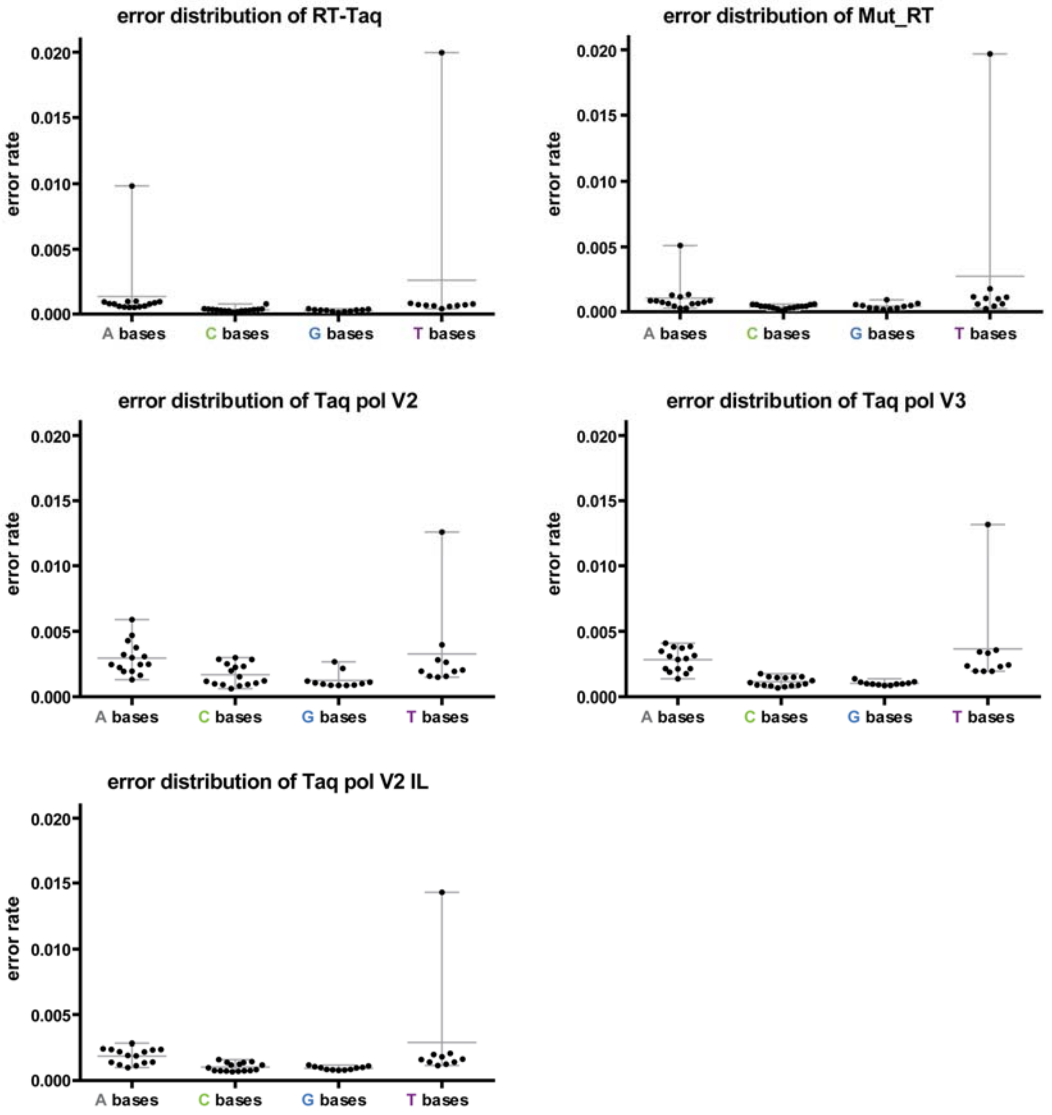

Figure S6: Error distribution of Taq pol variants while processing the SARS-CoV-2 RNA in the N1 region. Error rates (black dots) for each DNA pol were grouped separately according to their base identity and illustrated in a dot plot (errors from region without primer binding sites are included). Grey lines indicate the mean of each group and the range of the data points. Dot plots were prepared by using GraphPad Prism version 6.00 for Windows, GraphPad Software, La Jolla California USA.

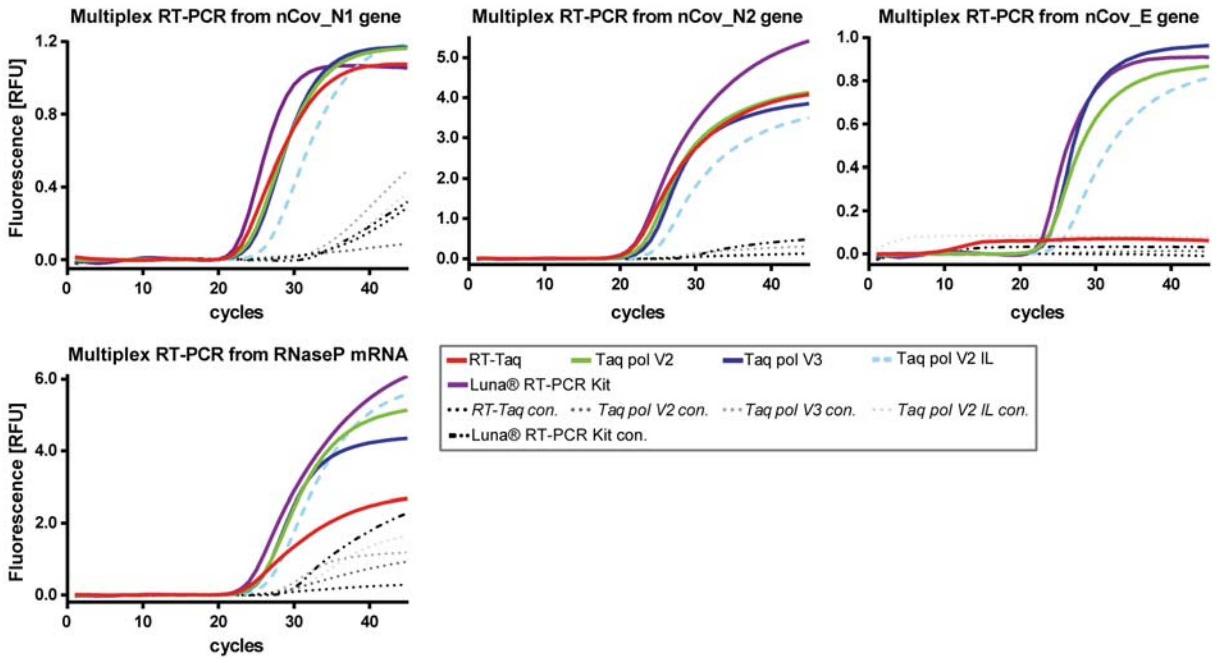

Figure S7: Amplification curves after multiplex RT-PCR from SARS-CoV-2 RNA and human RNase P transcript with comparison to Luna® Universal Probe One-Step RT-PCR Kit from NEB. 10.000 c/µL SARS-CoV-2 RNA and 1 ng/µL universal was used as template. 1 ng/µL universal human reference RNA was used to monitor amplification from the RNase P transcript. The genes nCoV_N1, N2 and E, as well as the RNase P transcript were analysed simultaneously (quadruplex RT-PCR). 120 nM purified Taq pol variants (as indicated) with 400 nM Taq pol aptamer or 1x Luna enzyme mix was used for catalysis (purple). The Luna Universal Probe One-step Reaction mix was used for each reaction. 670 nM primer, 170 nM TaqMan probe (for nCoV_N1/N2, RNase P) and 850 nM primer, 216 nM TaqMan probe (for nCov_E) were present in the reaction mix. Reactions were monitored by measuring the fluorescence from the 6-FAM dye (nCoV_N1), the Sun dye (nCoV_N2), the Texas Red dye (nCov_E) and the Cy5 dye (RNase P).

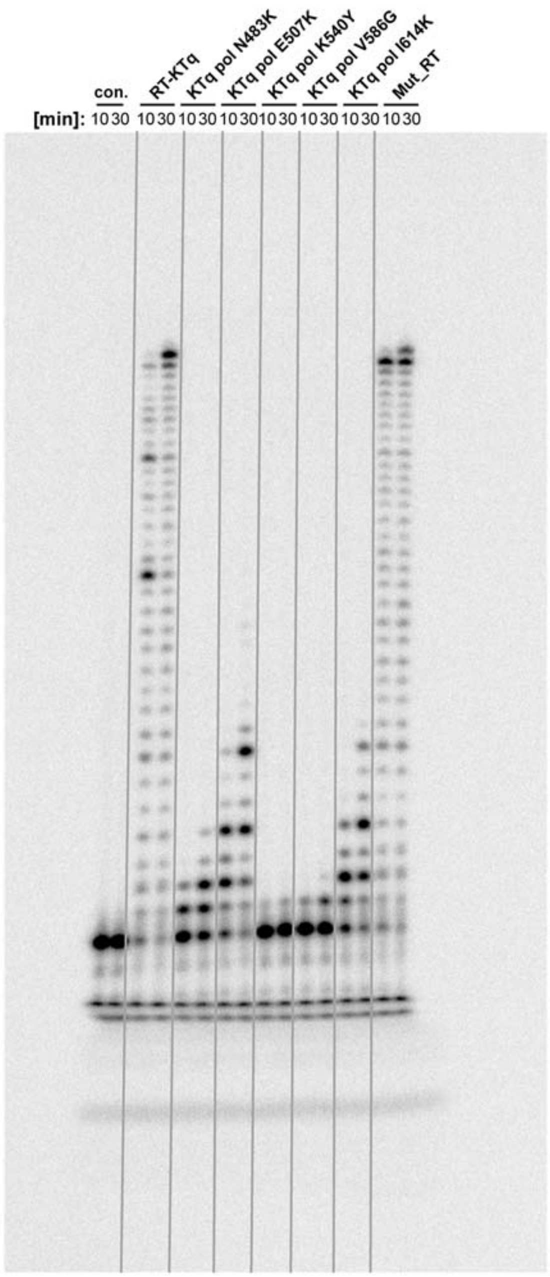

Figure S8: Original phosphor imaging scan of the primer extension with radioactively labelled primer from the artificial RNA oligonucleotide depicted in Figure S1 A. Imaging was done by usage of Thyphoon FLA 9000 (GE Healthcare) and Image Lab 6.1 (BioRAD).

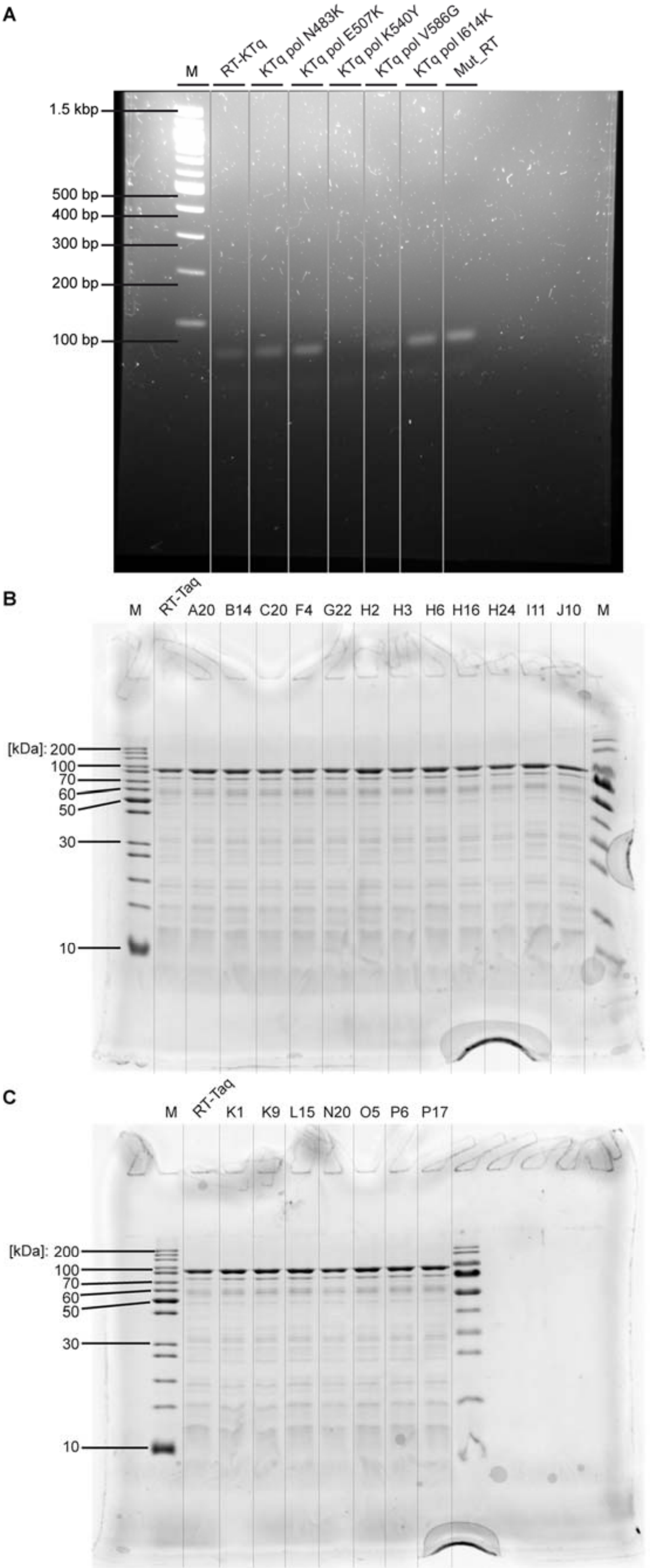

Figure S9: A) Uncropped agarose gel from Figure S1 C. Gel Doc XR+ Gel Documentation System (BioRad) and Image Lab 6.1 (BioRad) was used for imaging. B/ C) Uncropped gels from SDS-PAGE analysis of cell lysates containing Taq pol variants depicted in Figure S4 A. Amersham Imager 600 RGB (Ge Healthcare Life Sciences) and Image Lab 6.1 (BioRad) was used for imaging.

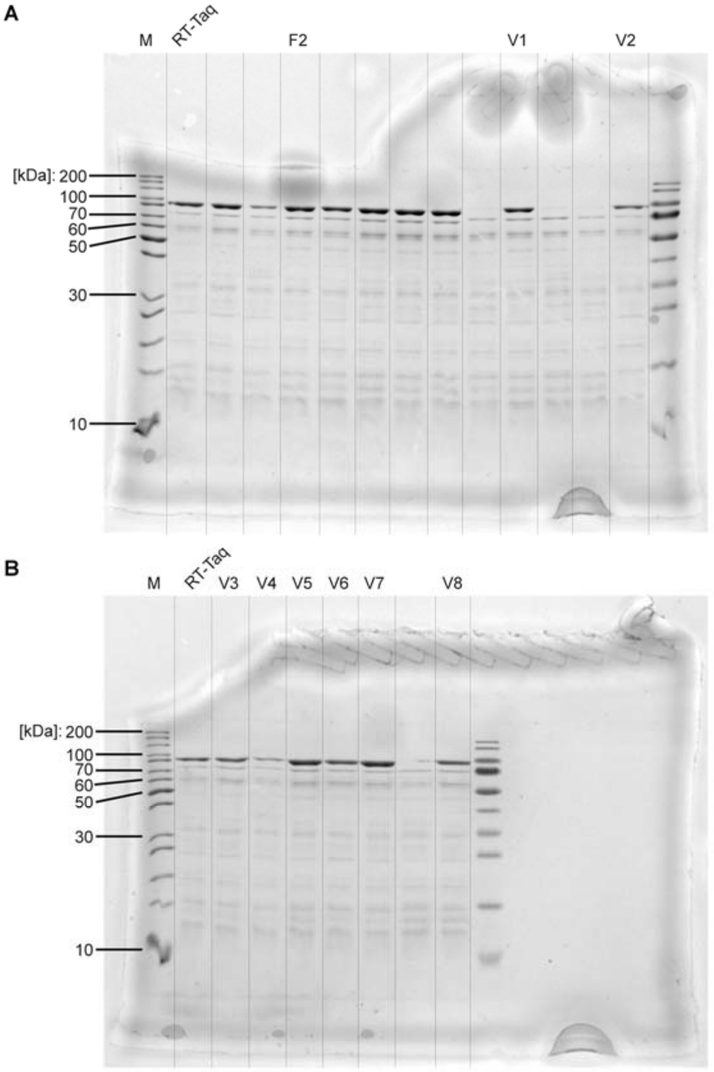

Figure S10: A/ B) Uncropped gels from SDS-PAGE analysis of cell lysates containing Taq pol variants depicted in Figure S4 A, B. Amersham Imager 600 RGB (Ge Healthcare Life Sciences) and Image Lab 6.1 (BioRad) was used for imaging.
